# Multi-sided platforms in competitive B2B networks with varying governmental influence – a taxonomy of Port and Cargo Community System business models

**DOI:** 10.1007/s12525-022-00529-z

**Published:** 2022-04-26

**Authors:** Ruben Tessmann, Ralf Elbert

**Affiliations:** Technical University of Darmstadt, Hochschulstraße 1, 64289 Darmstadt, Germany

**Keywords:** Taxonomy, Port Community System, Cargo Community System, Cluster Analysis, Business model, Multi-sided platform, L86, L91, M15, O3

## Abstract

Our knowledge on differences in business model characteristics of thriving and failing Multi-Sided Platforms in competitive B2B networks (B2B-MSP) and potential influences of increasing governmental involvement remains fragmented. This study develops a taxonomy to classify special B2B-MSP with varying governmental influence in the supply chain and transportation context, viz. Port and Cargo Community Systems (CS). Based on the classification of 44 international CS, we identify four archetypes using cluster analysis. The taxonomy provides practitioners with a differentiated view on the configuration options of CS business models including the involvement of governmental institutions, while the presented archetypes contribute an aggregated view of CS business models. The statistical analysis of our results provides initial explanatory approaches on CS business model dimension interdependencies, thereby laying the basis for a deeper understanding of sectoral and geographic differences of B2B-MSP and their diffusion dynamics as well as facilitating a higher contextualization of future research.

## Introduction

Seaports and airports around the world have been implementing Port and Cargo Community Systems (CS) to enable efficient information exchange as well as an extended service offer for their stakeholders (EPCSA, 2011; Moros-Daza et al., 2020). With technological advancement, the initial information exchange focus has broadened towards a coopetition and ecosystem scope, which involves a wider range of processes and stakeholders (Adaba & Rusu, 2014; Kenyon et al., 2018) on which ports heavily rely on during supply chain disruptions such as the worldwide Covid-19 vaccine distribution (Putzger, 2020b).

CS present a unique set of characteristics that distinguish them from other digital platforms and therefore justify a dedicated analysis. CS are digital multi-sided platforms (MSP) connecting competitive, international B2B networks (Wallbach et al., 2019) and can add, as such, to the limited body of literature concerning MSP in B2B contexts (Loux et al., 2020). As MSP, CS minimize the necessary interfaces while enabling a direct interaction amongst all involved stakeholders (Hagiu & Wright, 2015). Distinctively, deeply involved governmental stakeholders, such as legislators, port authorities, customs and police departments exert strong influence on the platform and its members. Commonly, governmental influence is not considered in the context of MSP and marketplaces (Bivona & Cosenz, 2021; de Reuver et al., 2018; Täuscher & Laudien, 2018). Additionally, CS are locally bounded in that they only address the stakeholders of ports in one country or even only one singular port. Lastly, in comparison to many of the successful and well-studied MSP examples such as Amazon or eBay, CS connect a wider range of stakeholders, viz. members of various supply chains, governmental agencies and value- added service providers such as banks and insurances (Rodon et al., 2008). Interactions and services offered through the CS are both consumed and provided by all members and therefore also have a peer-to-peer character.

A variety of studies has investigated the factors influencing the adoption and assimilation of CS (Rodon et al., 2008; Simoni et al., 2020; Wallbach et al., 2019), but we argue that a taxonomy of CS is needed in order to improve contextualization, comparability and clarity. Recent findings from IS and management literature suggest that platforms of the same general sector but with different business models in different geographical areas face different challenges (e.g., Cusumano et al., 2019; Gross et al., 2020; Li, 2019). Insights from one platform in its specific context might not be fully transferable to another. Practitioner reports on CS assimilation point into a similar direction (Gladiator, 2020). Accordingly, contextualization is relevant for CS studies (Moros-Daza et al., 2020), but also in general IS research on platforms (de Reuver et al., 2018; Robey et al., 2008). Comparability is also hindered by inconsistent terminology in the context of CS. For example, the term “single window” sometimes refers to a single point of entry of B2G communication (Heilig & Voß, 2017; Morton, 2018), but other authors use it to describe a Port Community System that offers a much wider range of services (Adaba & Rusu, 2014).

External disruptions, such as changing international circumstances which increase B2G communication requirements (e.g. Brexit) (Courea, 2020) or cyberattacks on the digital infrastructure with serious consequences (Warrick & Nakashima, 2020) show that modern ports are highly dependent on a seamless exchange of information and integration of digital services. In light of these developments, a lacking adoption of CS as central information platforms in developing countries and less frequented transport network nodes such as dry ports (Moros-Daza et al., 2020) paired with a CS adoption literature body that does not offer concise insights into contextualized facilitators and barriers due to hampered comparability of studies is debilitating. In both a scientific and practical discussion of CS, it is important to consider their different characteristics to better understand how they are best designed and unfold in practice. At the same time, existing taxonomies of digital platform business models are not directly applicable to CS due to their special attributes, such as a sometimes- strong governmental influence or geographic boundedness. Accordingly, we want to investigate the characteristics differentiating CS business models to build a CS-specific taxonomy. By using a CS-specific taxonomy, interested parties can better identify other platforms using similar business models on the market, the difficulties they face as well as potential growth opportunities. MSP research can benefit from a detailed view on CS business models as the contextualized insights from B2B-focused, locally bounded MSPs with varying governmental influence can support a better understanding of MSPs as a comprehensive concept. All three contexts are relevant for that matter, as it is firstly still not clear why B2B-focused MSPs have not gained the same momentum that their B2C or C2C counterparts have (Riemensperger & Falk, 2020)**,** as secondly de Reuver et al. (2018) already asked which role boundaries play for digital platforms and only a limited number of platforms that are bounded locally exists and lastly, as MSP research rarely investigates the implications and effects of governmental influence, which is gaining importance due to regulators slowly catching up with the special conditions of MSP markets (Evans, 2008; Reck, 2021). Therefore, we formulate the following research question:

- **RQ1**: Which dimensions and characteristics of Port and Cargo Community Systems business models exist and can any of these further the extant knowledge on MSP business models?

To answer RQ1, we draw on and synthesize research on CS and business models of adjacent domains, as well as the results of an analysis of 44 international CS. We want to showcase the usefulness of the developed taxonomy by extending the conceptualization of CS platforms and help to overcome the ambiguity of terminology in the field by distinguishing archetypes of CS based on the previously deduced characteristics. By “archetype” we refer to a representative example of a particular type of CS, i.e., what typically distinguishes different types of CS. Accordingly, our second research question is:

- **RQ2**: Which archetypes of Port and Cargo Community System business models can be distinguished?

We thereby answer the call of Moros-Daza et al. (2020) to add to the limited body of holistic CS research studies by providing a taxonomy which allows to describe CS as well as their context in a structured way and therefor acts as a “*theory for analyzing”* (Gregor, 2006). This can be the foundation of future contributions to develop more advanced theories which explain, predict or give design and action advice (de Reuver et al., 2018; Gregor, 2006). Furthermore, we present archetypes of CS business models which can serve researchers and practitioners in comparing existing CS amongst each other and to other digital platforms based on their characteristics. This can support future decisions on CS and other platform developments (de Reuver et al., 2018). With our results we also lay a basis for answering the call of de Reuver et al. (2018) for a deeper understanding of sectoral and geographic differences in the success factors of digital platform assimilation. Additionally, we support future research in the field to follow Robey et al. (2008)’s suggestion that “Inter- organizational systems’ (IOS) characteristics” should be explored in IOS adoption research, where the context in which an IOS is adopted as well as the governance arrangements are seen as particularly relevant.

The article proceeds as follows: First, we describe and discuss the domain background of CS as well as extant taxonomies on platform and digital business models. Next, we develop a taxonomy for CS business models based on the procedure proposed by Nickerson et al. (2013). Then, we use the developed taxonomy to classify the business models of 44 international CS, conduct a cluster analysis and build archetypes that represent typical CS business models. Finally, we discuss our results, outline the implications and limitations, and suggest further research.

## Domain background

### Community Systems as multi-sided platforms in competitive B2B networks with governmental influence

CS are geographically bounded, digital, multi-sided platforms, enabling more efficient and effective business-to-business (B2B) and business-to-government (B2G) communication (Chandra & van Hillegersberg, 2019; Moros-Daza et al., 2020; Srour et al., 2008). Additional services are realized and offered through a modular architecture of CS (e.g., Carlan et al., 2016; Mayanti et al., 2020; Simoni et al., 2020; Wallbach et al., 2019). Depending on the specific location of CS, they are referred to as “Port Community System” (PCS) in the context of seaports and “Cargo Community System” (CCS) or “Airport Community System” (ACS) at airports (Carlan et al., 2016). While there is an abundant research body on PCS (cf. Moros-Daza et al., 2020), CCS/ACS have received less attention (e.g., Chandra & van Hillegersberg, 2019; Christiaanse & Damsgaard, 2006; Wallbach et al., 2019). As briefly mentioned in the introduction, currently, a certain ambiguity and conceptual overlap exist between the terms “Community System” and “Single Window” (e.g., Moros-Daza et al., 2020). The International Port Community System Association (IPCSA) stresses the interconnectivity of the various (private) firms of a port community as a key characteristic of CS (Morton, 2015). Accordingly, we excluded all those locally bounded platforms labeled “single window”, which solely cover B2G, G2B and G2G interactions, as we consider them to fall under the research field of e-government (cf. e.g., Abramson & Morin, 2003; Davison et al., 2005; Silcock, 2001).

While CS have been described as digital MSP before (Moros-Daza et al., 2020; Wallbach et al., 2019), they can be considered a special case of MSP as they connect a wide variety of distinct groups, from the various cargo transport network actors, software developers, banks and insurances (B2B-connections) to governmental agencies such as customs, port authorities, and others (B2G- and G2G-connections) (Rodon et al., 2008; Wallbach et al., 2019). CS stakeholders regularly face network externalities (Wallbach et al., 2019), i.e., positive or negative effects that arise from other platform participants either from their own or another side (cf. Evans & Schmalensee, 2016; Parker et al., 2016 for the distinction between positive and negative, same-sided or cross- sided network effects).

CS are set in medium to highly competitive B2B networks, depending on the local circumstances. Wallbach et al. (2019) describe the ACS of Frankfurt, Germany airport to be a highly competitive B2B network based on the distinction of competitive models by Farahani et al. (2014). Wallbach et al. (2019) divide B2B markets into three distinct groups based on five characteristics, viz. market structure, market share/barriers, regulation, product and service characteristics and governance structure. Due to the air cargo market at the airport of Frankfurt being polypolistic, with low market shares per stakeholder, regulation being imposed by the market rather than the government, the service characteristics being interchangeable and the network being governed by a broader community, it falls in the highly competitive category of B2B networks (Wallbach et al., 2019). While some evidence from PCS seems to support this categorization also for seaports (e.g., Rodon et al., 2008), other authors point out the crucial role that governmental agencies such as the port authority or customs have, especially in ports with a non-privatized management model (e.g., Adaba & Rusu, 2014; Chandra & van Hillegersberg, 2018; Damsgaard, 1999; Gustafsson, 2007). Given that in such cases a seaport is rather lead-organization governed and has regulations that are at least partially imposed by government actors, seaports can also be viewed as conventional or medium competitive B2B networks. In contrast to other B2B MSP (e.g. Agarwal & Brem, 2015; Kumar, 2019) and B2C MSP examples, such as Amazon or eBay (Cusumano et al., 2019; Gawer, 2014), which might experience regulation from governments at some point in their existence (Reck, 2021), CS commonly face a long-lasting and strong influence by governmental agencies, as those are commonly part of the directly involved stakeholders (Chandra & van Hillegersberg, 2018).

Moreover, CS are locally bounded, which potentially offers interesting insights compared to internationally active platforms (de Reuver et al., 2018). The individual CS needs to be adapted to local conditions, therefore a one-size-fits-all strategy of implementing the same CS at every port around the world has proven to be difficult (Baron & Mathieu, 2013; Moros-Daza et al., 2016). The majority of CS is used on a local port or – at best – a country level (Baron & Mathieu, 2013).

### Platform and digital business model taxonomies

Publications discussing taxonomies cover a highly diverse set of topics, and we want to focus on those that are loosely related to our research question, i.e., taxonomies on platform or marketplace business models and digital business models. To identify such taxonomies, we conducted a semi-structured literature search utilizing the procedure described by Durach et al. (2017). A detailed description of the keywords, including and exclusion criteria can be found in the appendix. Most business model taxonomies are based on similar basic frameworks, for example on the practitioner-oriented business model canvas (Osterwalder & Pigneur, 2010) and extend or enrich the respective dimensions according to the specific context (e.g., Passlick et al., 2021; Schoormann et al., 2016; Weking et al., 2020). To structure the evaluation of existing taxonomies, we utilize three initial criteria based on the differentiating characteristics of CS as described above, viz. a B2B focus, a more or less direct influence of governmental stakeholders and the local boundedness of the platform. Table 1 presents a non-exhaustive overview of taxonomies in the context of platform or digital business models and if they address any of the three initial criteria identified in the context of CS. Although we looked specifically for taxonomies on platform and digital business models that cover local boundedness of such, we were not able to identify any. Most platform business models are assumed to particularly not be bounded locally, one of the reasons for de Reuver et al. (2018) recently asking whether geographic boundaries matter.

**Table 1:** Overview of taxonomies in relation to key aspects of CS and themes of platform and digital business models covered

Source Perspectives	Key aspects of CS covered			Business model themes covered			
	B2B focus	Governmental influence	Local boundedness	Stakeholder ecosystem	Value creation	Platform architecture & organting model	Value capture
Remane et al. (2016)					x	x	x
Bock and Wiener (2017)					x	x	x
Täuscher and Laudien (2018)				x	x	x	x
Blaschke et al. (2019)				x	x	x	
Hodapp et al. (2019)					x	x	x
Passlick et al. (2021)	x	(x)		x	x		x
Weking et al. (2020)	x			x	x	x	x
Abendroth et al. (2021)	x			x	x	x	x
Bivona and Cosenz (2021)					x		x

Some authors argue, that digital platforms and their respective business models are not directly comparable to traditional companies and non-digital platforms as they offer some special characteristics (cf. de Reuver et al., 2018). For example, in contrast to traditional business models, digital platforms regularly create at least parts of their value through increased usage (Vargo & Lusch, 2008). The VISOR framework (El Sawy & Pereira, 2013) for digital business models, tries to account for these special characteristics as it introduces new meta- dimensions, i.e., on top of the commonly used Value proposition and Revenue model dimensions, also Interface, Service platform and Organizing model. Hodapp et al. (2019) as well as Remane et al. (2016) and Remane et al. (2017) utilize the VISOR framework for their respective taxonomies. Remane et al. (2016) build a taxonomy for car sharing platforms, which has a strong B2C and C2C focus and does not cover any of the three key characteristics of the CS context. Similarly, Hodapp et al. (2019)’s taxonomy for Internet of Things platform business models, which can comprise both B2C and B2B interactions, is too specific to be useful for classifying CS on the one hand and also does not account for the governmental influence and local boundedness of these platforms. Bock & Wiener (2017) choose a different approach, as they aim to develop a general, non-domain-specific taxonomy on digital business models. They identify “digital offering”, “digital experience”, “digital platform”, “data analytics” and “digital pricing” as the dimensions of their taxonomy (Bock & Wiener, 2017). Given the generality of this particular taxonomy, CS can be located on it, but a distinction of different CS aspects seems difficult. Take for example the “digital offering” dimension, which is divided into five characteristics, viz. digital products, digital services, human services, complementary digital services and physical products with embedded digital technologies. The vast majority of CS will only offer digital products (e.g., data (Rodon et al., 2008)) and digital services (e.g., track and trace services inside the port (Simoni et al., 2020)), as none of the other characteristics can be applied in this context. Therefore, we conclude that the digital business model taxonomy of Bock & Wiener (2017) is not specific enough for our context, given that it neither accounts for the B2B focus of CS nor the role governmental actors or the implications of the local boundedness of the ecosystem. Passlick et al. (2021) develop a taxonomy for predictive maintenance business models, which are not solely digital business models but are closely related to the Internet of Things, and we therefore include their taxonomy in our overview. The taxonomy focuses on B2B relationships and considers, at least partially, the influence of governmental actors, even though they are only seen as potential customers not as influencing stakeholders. Overall, the taxonomy of Passlick et al. (2021) is too specific for our context, as it neither focuses on platform nor solely digital business models and also does not account for regional limitations of companies’ business models.

Täuscher & Laudien (2018) create a taxonomy on the key business model characteristics of (digital) marketplaces. They aggregate 14 dimensions into three meta-dimensions, viz. value creation, value delivery and value capture, but the resulting framework is again too general for our context. The “Marketplace participants” identified cover C2C, B2C and B2B, so the taxonomy is not B2B-centric (Täuscher & Laudien, 2018). In contrast to the previously presented taxonomies, Täuscher & Laudien (2018) do not focus on the platform architecture, i.e., how the interface is designed and what the technological foundations of the platform are. Additionally, governmental stakeholders and their influence as well as the local boundedness of the platforms are not covered by Täuscher & Laudien (2018). Similarly, the framework designed by Bivona & Cosenz (2021) to assess the business models of multi-sided platforms is designed and tested only in a B2C context and is therefore not directly transferable to our context. Abendroth et al. (2021) create a context-specific taxonomy on B2B co-creation platforms. They identify three meta-dimensions, viz. value creation, platform architecture and actor ecosystem and thereby stress the importance that the architecture and ecosystem have in MSP. Their taxonomy is too specific for our context, though, because co-creation is not the sole focus of CS (Moros-Daza et al., 2020). Blaschke et al., (2019) are even more specific, as they focus their taxonomy on the platform architecture dimension. Finally, Weking et al. (2020) develop a taxonomy of industry 4.0 business models, which covers a wide variety of firms. Accordingly, they base their taxonomy on basic components of existing business model frameworks (Foss & Saebi, 2017; Osterwalder & Pigneur, 2010; Saebi et al., 2017; Teece, 2010). Their final taxonomy is built on the five meta-dimensions of target customers (Who?), Value Proposition (What?), Value Chain (How?), Key Elements (How?) and Value Capture (Why?), with the key elements meta-dimension comprising a platform dimension (Weking et al., 2020).

In summary, extant research on business model taxonomies provides only limited guidance in terms of what characterizes a CS as well as what distinguishes different CS from each other, which is an extant research gap as it hampers the extraction of contextualized insights for these multi-sided B2B platforms with strong governmental influence. Existing taxonomies from adjacent domains, such as platform or marketplace research are either too domain-specific or too generic to be useful for CS research, as they fail to account for these platform’s special characteristics. Nonetheless, all of the described taxonomies can build the theoretical basis for our taxonomy on CS.

## Research methodology

### Rationale and data collection

As CS are an emerging and ever-evolving phenomenon, there is little guidance for its comparative analysis and accordingly its context-specific design (de Reuver et al., 2018; Moros-Daza et al., 2020). Without objectified criteria, a structured analysis of CS is hardly possible. For the first step of our research, we require a system of measurable characteristics to structure and simplify a complex collection of MSP. In consequence, we see a taxonomy development to be an appropriate approach to provide a structuring that can be used to subsequently analyze the connections between CS characteristics to discover archetypes and thereby build a “*theory for analyzing”* (Gregor, 2006) which can act as the foundation of future contributions in the field.

For the identification of relevant objects, i.e., Community Systems, for taxonomy building and evaluation, we followed a three-stage approach, following the call of Vom Brocke et al. (2009) for more rigor in the reporting of literature review procedures. As the first step, gaining a baseline sample of relevant CS, we build on a recent collection of 48 Port Community Systems (Moros-Daza et al., 2020). In the second step, we identify further CS by a literature search extending our preliminary sample to 77 CS. We only include CS in our final sample of 44 CS, for which we could enrich the available information to a satisfactory level, i.e., with at least one peer-reviewed source and one non-peer reviewed source or three non-peer reviewed sources with relevant information on characteristics of the respective CS. A detailed description of the data collection process can be found in the Appendix.

### Taxonomy development

**Fig. 1:**
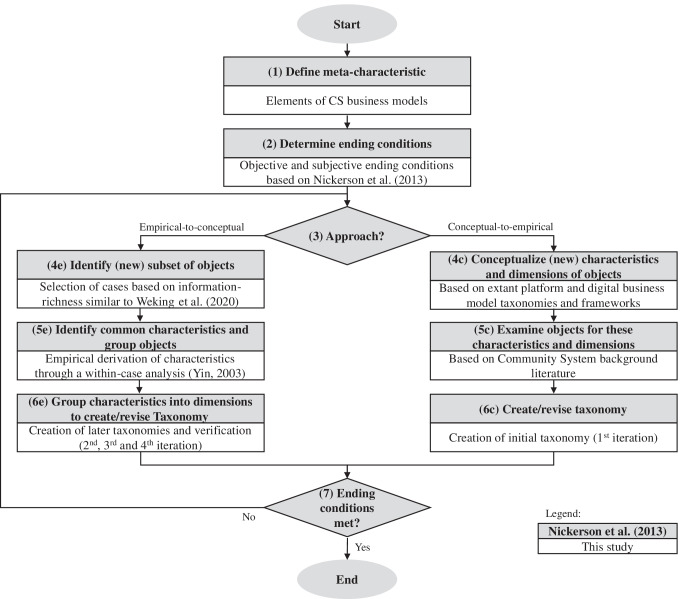
Taxonomy development method according to (Nickerson et al., 2013) adapted from (Szopinski et al., 2020)

Figure 1 depicts the iterative taxonomy development process by Nickerson et al. (2013) which consists of seven steps and has been applied widely since its publication (e.g., Gimpel et al., 2018; Passlick et al., 2021; Szopinski et al., 2020; Weking et al., 2020) and can therefore be considered an accepted research method. The first step is the definition of a meta-characteristic, which is the foundation of all further steps and due to the iterative nature of the method, ending conditions have to be defined (Nickerson et al., 2013). For each iteration (i.e., steps 3 to 7), one of two approaches has to be chosen: either an empirical-to-conceptual approach, which is inductive and should be used in case of availability of sufficient real-world data or a conceptual-to-empirical approach, which is deductive and is supposed to leverage (existing) knowledge of the authors and from literature related to the meta-characteristic (Nickerson et al., 2013). The taxonomy is revised after each iteration and terminates once the ending conditions are met. The following paragraphs describe the iterative process that led to our taxonomy of CS business models.

- *(1) Definition of meta-characteristic:* Similar to the approach of Weking et al. (2020), we choose four meta-characteristics, or “perspectives” (Gimpel et al., 2018), based on the previously described domain background (see Table 1). Firstly, we capture who is involved in CS platforms with the “Stakeholder ecosystem” (Abendroth et al., 2021) perspective. This is similar to the target customer (Weking et al., 2020) or customer segment and clients (Passlick et al., 2021) perspectives as well as the key partners and customer segments categories (Osterwalder & Pigneur, 2010) of other business model related taxonomies and frameworks. In the context of CS, stakeholders are commonly both partners and customers (Moros-Daza et al., 2020; Rodon et al., 2008). Secondly, we examine what creates value for the stakeholder ecosystem of a CS platform (“Value creation” (Abendroth et al., 2021; Weking et al., 2020)). “Value creation” is used across various taxonomies (e.g., Hodapp et al., 2019; Passlick et al., 2021) as well as business model frameworks (e.g., Foss & Saebi, 2017; Osterwalder & Pigneur, 2010; Teece, 2010) and is one of the dimensions of the value delivery perspective of the platform business model’s taxonomy of Täuscher & Laudien (2018). Thirdly, to describe how the CS platform is set up technologically and organizationally, we incorporate the “Platform architecture & organizing model” (Abendroth et al., 2021; Remane et al., 2016) perspective. It is intended to integrate several overlapping meta-characteristics and dimensions from platform and digital business model taxonomies. Those are, for example, the “core” and “infrastructure” dimensions from Blaschke et al. (2019), the “platform type” dimension from Täuscher & Laudien (2018), the “digital platform” and “data analytics” dimensions of Bock & Wiener (2017), the “Interface”, “Service platform” and “Organizing Model” dimensions of El Sawy & Pereira (2013) and also some dimensions of the “Key elements” perspective of Weking et al. (2020). Finally, the fourth perspective is “Value capture” (Foss & Saebi, 2017; Täuscher & Laudien, 2018; Weking et al., 2020) which describes why and how the platform is financially viable. It summarizes the “revenue model” perspective of El Sawy & Pereira (2013), the “digital pricing” dimension of Bock & Wiener (2017) and the “revenue stream” dimensions of Abendroth et al. (2021) and Osterwalder & Pigneur (2010)
- *(2* + *3) Ending conditions & Selected approaches*: We adopted the objective and subjective ending conditions from Nickerson et al. (2013) and report on those in Table 5 in the appendix. On the one hand, there are five subjective ending conditions, i.e., when the taxonomy is concise, robust, comprehensive, extendible, and explanatory, one can end the iterative process. On the other hand, eight objective ending conditions indicate that the iteration process can be ended. When all objects that have been identified for the taxonomy development have been examined, when no objects were merged with similar object or spilt into multiple objects in the last iteration, at least one object is classified under every characteristic of every dimension, no new dimensions or characteristics were added, merged or split in the last iteration, every dimension is unique and not repeated and every characteristic is unique within its dimension as well as each combination of characteristics is unique and not repeated, the iteration process is terminated. We iterated through a total of one conceptual-to-empirical cycle (1^st^ iteration) and three empirical-to-conceptual cycles (2^nd^ – 4^th^ iteration), as all ending conditions were met after the 4^th^ iteration. We started the iteration process with the conceptual-to-empirical cycle, as we wanted to leverage knowledge from extant literature on both CS as well as taxonomies from the platform and digital business model context, to build a solid baseline for our taxonomy.
- *(4c – 6c) First iteration (conceptual-to-empirical)*: Based on the four meta-characteristics described above, we derived dimensions and characteristics from extant literature on platform and digital business model taxonomies as well as CS background literature. As for the stakeholder ecosystem perspective we started with three dimensions, namely “market” (Täuscher & Laudien, 2018; Weking et al., 2020), “geographic scope” (Abendroth et al., 2021; Täuscher & Laudien, 2018), and “modes of transport”, which was derived from the common distinction of CS based on their distinctive transportation mode (Carlan et al., 2016). Communication and value- added services were added as they are central aspects of many CS’ value propositions (Moros-Daza et al., 2020; Rodon et al., 2008). We initially included a “review system” dimension (Täuscher & Laudien, 2018), but had to omit it as we could not find any evidence to support it in the context of CS. We added the “platform origin” dimension as a CS can either be designed uniquely or based on another, existing CS (Baron & Mathieu, 2013; Moros-Daza et al., 2020). The “interface” dimension is taken from the VISOR framework (El Sawy & Pereira, 2013) and “data analytics” from Weking et al. (2020). The “Role of governmental actors” dimensions is deducted from strong involvement of governmental actors in CS. Both decisional openness and complementor openness have been adopted from Abendroth et al. (2021), as some CS seem to develop into that direction lately (Elbert & Tessmann, 2021). Given the high involvement of governmental stakeholders, the business objective of the CS operator was added as a dimension, as some companies operate on a not-for-profit basis (Chandra & van Hillegersberg, 2018, p. 58). “Funding”, “funding continuity” and “payment model” have been chosen as dimensions based on the various value capture or revenue model dimensions of extant adjacent taxonomies (Remane et al., 2016; Täuscher & Laudien, 2018; Weking et al., 2020). The initial characteristics have been transferred from their respective dimension sources and adapted to our context in step 5c based on the CS background literature.
- *(4e – 6e) Second through fourth iteration (empirical-to-conceptual)*:
- The 44 sample CS were divided into three groups (i.e., sub-samples) as shown in Appendix Table 6, based on information richness similar to the approach of Weking et al. (2020), i.e., based on the number and content of sources that could be retrieved for a respective CS. In the second iteration, based on Sample A, we adapted the characteristics of several dimensions (see Table 2). We also added a total of three dimensions in the second iteration, viz. “Extended services” in the “Value creation” perspective, as well as “Data security” and “Data governance” in the “Platform architecture and organizing model” perspective. The third iteration, based on Sample B, led to a change of the characteristics in five dimensions (see Table 2) and no further dimensions had to be added or altered. The fourth and final iteration, based on Sample C, did not lead to any further changes.
- *(7) Ending conditions, empirical and theoretical evaluation:*
- With the fourth iteration, all ending conditions were fulfilled (see Steps 2 and 7 in Figure 1 in conjunction with Appendix Table 5). For a consistent and exhaustive identification of dimensions and characteristics, the first author coded all case examples within one week, while the second author proclaimed authentic dissent or took the role of a devil’s advocate by suggesting alternative explanations and raising critical questions regarding the taxonomy (Eisenhardt, 1989; Nemeth et al., 2001). This was done to uncover potential deficiencies and to scrutinize assessments in order to improve the quality of the taxonomy, similar to the approach of Szopinski et al. (2020).
- We deviate from Nickerson et al. (2013)’s objectivization of their subjective ending condition of conciseness, i.e., using a maximum of nine dimensions for a taxonomy. Therefore, we additionally utilized a similar approach to that of Weking et al. (2020), who also created a taxonomy with more dimensions, to empirically evaluate our taxonomy in a two-step approach. As first and main evaluation step, we organized a virtual meeting and conducted a confirmatory focus group with six participants plus the first author who acted as the moderator (Tremblay et al., 2010). The meeting was not in-person due to the current Covid19 pandemic. The six participants were selected based on their research or work focus on digital business models and previous experience in the field of taxonomy creation or sea- and airports (e.g., management consulting background). The meeting took a total of 60 minutes, where the first ten minutes consisted of the introduction and problem description and the remaining 50 minutes were spent reviewing and discussing the taxonomy. This led to minor changes in three of the taxonomy’s dimensions. Specifically, some of the characteristics were renamed for clarification purposes (see Table 2). The focus group discussions also showed the necessity of a more concise depiction of the characteristics, while the overall large number of dimensions was seen as uncritical.
- Accordingly, we deviate from Nickerson et al. (2013) in that we do not use mutually exclusive characteristics for all dimensions in Table 2, similar to Gimpel et al. (2018). Exemplarily, take the “Market” dimension, where a single CS can cover multiple markets (e.g., B2B & B2G). Listing all (possible) combinations of the six characteristics in Table 2 would make them mutually exclusive (cf. for example Passlick et al. (2021)’s “Deployment channel” dimension), but less easily understood. In the second step, to verify the applicability and usefulness, we used an exploratory cluster analysis to identify archetypes of CS.

**Table 2:**
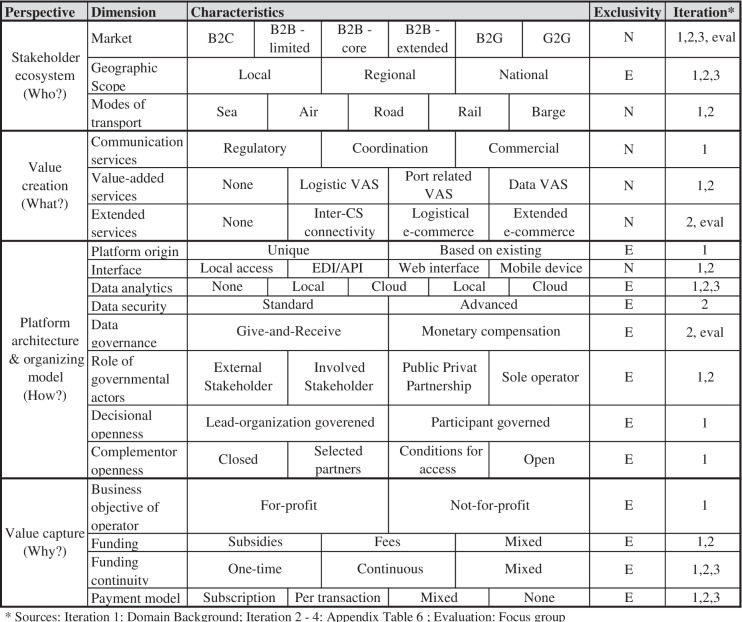
Taxonomy of Community System business models, including exclusivity column (E – exclusive, i.e., only one characteristic observable with one object; N – non-exclusive, i.e., multiple characteristics observable with one object) and iteration column, specifying, in which iteration the respective dimension was altered

## Taxonomy of Community Systems business models

### Stakeholder ecosystem

Firstly, the “Market” dimension accounts for the potential connection of various businesses (B2B), the communication between businesses and governmental agencies (B2G), and amongst governmental actors (G2G), as well as the interaction with consumers (B2C, e.g., arrival updates for ferry passengers (Di Vaio & Varriale, 2020)). We define a core-group of private business stakeholders, viz. carriers, line agents, forwarders, terminal operators, custom’s brokers, shippers, consignees, carrier inland operators, and if they are private companies, the port authority, developers and the CS operator (cf. Fig. 5 in (Chandra & van Hillegersberg, 2018, p. 62)). Accordingly, at an airport, airlines are part of the core group instead of sea carriers. A limited group of stakeholders is any subset of the core group and the extended group has additional stakeholders involved in the CS, such as banks and insurances (Adaba & Rusu, 2014; Rodon et al., 2008). A CS can address different geographic scopes. It can be entirely local, i.e., focused only on the respective port, it can be regional in that it includes the hinterland of a port or national if it is applied in a country. In the latter case, the same platform is commonly used in multiple ports. The third dimension defines the modes of transport that a respective CS covers with its services, viz. sea, air, road, rail, barge and any combination of these.

### Value creation

The most basic services offered by a CS are communication services which can be regulatory, i.e., documents, such as customs manifest, ISPS declaration and others, coordinative, such as pre-notifications, shipment instructions and more or commercial, such as invoices or container release confirmations. On top of these communication services, certain value-added services can be identified. We categorized the multitude of available value-added services into three categories. Logistical value-added services comprise, for example, track and trace services, as well as warehousing and truck appointment services. Furthermore, we also summarized compliance services such as services related to dangerous goods and services that (actively) support CS stakeholders in submitting regulatory documents compliantly, as logistical value-added services. The second category of value-added services are port-related. On the one hand, these are services that support the port management in operating the port more safely and efficiently. On the other hand, these are services that support other stakeholders regarding port processes. Some CS include navigational features (waterways or roads), the payment of port dues directly through the CS, or physical safety and security features, such as live access restrictions through the CS for certain non-public areas of a port. Data related value-added services are the last category and comprise services such as data warehousing or business process optimizations, i.e., identifying optimization potentials for stakeholders based on the exchanged information.

Lastly, “Extended services” were identified as part of our empirical-to-conceptual iterations. The first extended services category is the Inter-CS connectivity. Some CS recently connected amongst each other, so that data could be exchanged between various international CS seamlessly as part of the “Network of Trusted Networks” initiative (IPCSA, 2020). Secondly, we found logistical e-commerce services, that go beyond logistical value- added services, in that users (e.g., a freight forwarder) could book logistical services for cargo through the respective platform itself, such as a last-mile delivery from a specialized inland carrier. Extended e-commerce services are services that can be bought through the platform, similar to an app-store, which cover non-logistical services and products, such as financial or insurance related services.

### Platform architecture & organizing model

The taxonomy distinguishes whether the digital platform, which is the technological backbone of the CS, is created uniquely or based on an already existing CS (e.g., PMAC, 2017) and which interfaces CS use. Some CS still rely on local access facilities (Long, 2009, p. 66) for companies that cannot send data electronically. Otherwise, stakeholders can access the CS directly through their own computer network (EDI/API), or indirectly through a web page or mobile device app. Depending on the scope of the value creation perspective, certain data analytics are an essential part of the platform. Our taxonomy distinguishes those based on where the data is stored, i.e., locally or decentralized (cloud), and if it uses common statistical approaches or more advanced big data or artificial intelligence (AI) based methods. Data security measures are distinguished based on the methods used to secure stakeholder data. An advanced security level indicates that multiple physical and virtual methods, e.g., firewalls, encryption, audits, server monitoring and duplication of databases are combined with modern technologies such as blockchain. As data sharing is essential in a CS, the taxonomy distinguishes how stakeholders are encouraged to share data, i.e., how the data governance is set up. It can be based on a give-and-receive scheme, i.e., stakeholders can only use the CS if they share data themselves or a monetary compensation can be implemented, where the provider of valuable data is compensated for sharing it (Lievens, 2017; Moyersoen, 2019). Governmental stakeholders can take different roles within a CS, from being external to it (e.g., Cheng & Wang, 2016) to being the sole operator. “Decisional openness” distinguishes whether the CS is governed solely by its lead-organization or more openly by its participants. Lastly,” Complementor openness” describes how open the CS is to co-developers.

### Value capture

The first dimension of the value capture perspective distinguishes the business objective of the operator. Especially through the involved governmental actors, not all operators are for-profit companies. Accordingly, funding can come from subsidies, fees or a mixture of both. Funding can come as a one-time lump sum or continuous or mixed. Lastly, the payment model can be based on a subscription, can be per transaction handled by the CS or can be a mixture of subscription, per-transaction, per user and per transport-unit handled. Also, no payment model is possible in case the stakeholders do not pay for the usage of the CS, as it is solely funded by subsidies.

## Taxonomy application—Community System archetypes

### Community System archetypes

For a better understanding of different CS business models and to verify the applicability of our taxonomy, we use a cluster analysis to identify relevant CS archetypes. We thereby make use of a mixed-methods approach (Kelle, 2006). In the appendix, a detailed description of our clustering method can be found, which accounts for three special conditions we found in our context, viz. categorial data, an overall small data set of only 44 CS and lastly, some missing data for the “Funding”, “Funding continuity” and “Payment model” dimensions of our “Value capture” perspective. To account for these special conditions, we utilize a distance measure that was specifically designed for categorical data (*Lin* similarity measure (Boriah et al., 2008)), use statistical clustering methods that are robust even for small data sets (complete linking hierarchical clustering (Šulc & Řezanková, 2019)) and apply a state-of-the-art multiple imputation method (Basagaña et al., 2013) to account for missing values and thereby minimize the effect of missing values on the results.

The cluster analysis results suggest the distinction of four clusters which means that the collected data indicates that four CS archetypes can be differentiated. Table 3 shows the characteristic distribution per dimension within each of those four archetype groups in comparison to the mapping of the entire CS sample (44). As we cannot describe and discuss every dimension and characteristic in detail due to space limitations, we will first provide a short description of each of the four archetype groups and then present some of the results more in-depth based on an analysis of contingency tables.

**Table 3:**
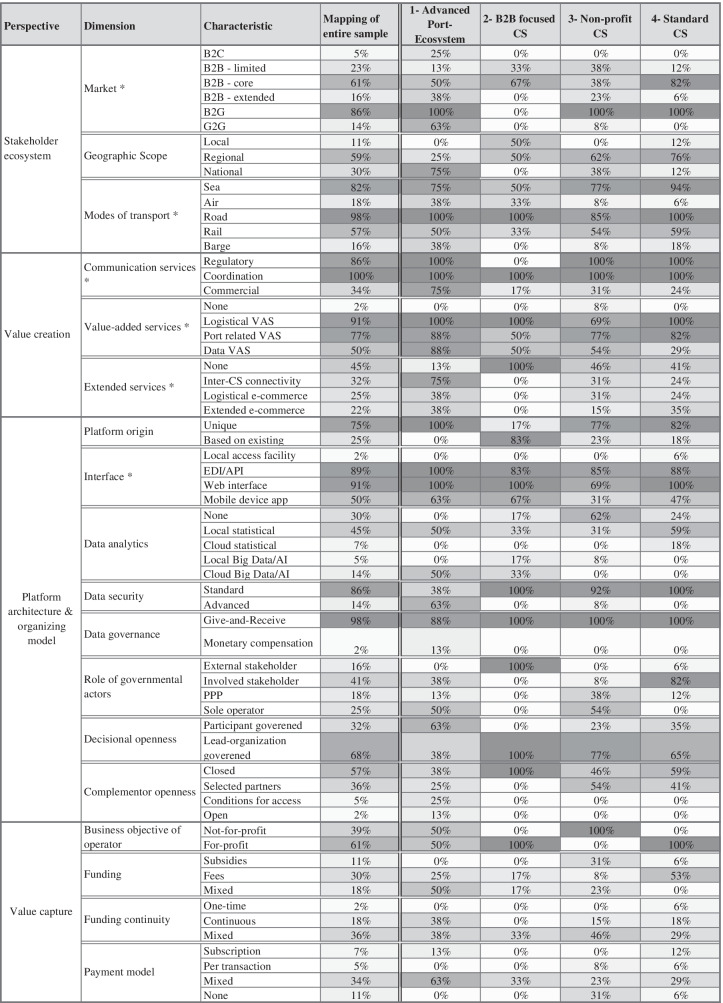
Results of the cluster analysis

Non-exclusive dimensions have been marked with an asterisk. The “Funding”,“ Funding Continuity” and “Payment model” dimensions have been reported based on the non-imputed (WOM) data, i.e., the column sums per category are < 100% when data is missing

We labeled the first archetype group as “*Innovation-oriented port eco-systems*” as these eight CS address the most extended stakeholder group, involving B2C and G2G as well as extended B2B markets, i.e., also including stakeholders such as banks and insurances and act mostly on a national, rather than a local or regional level. Also, in all dimensions of the “value creation” perspective they have the widest functional scope, such as regularly including commercial communication as well as all identified types of value-added services. They are also early adopters of extended services, such as inter-CS connectivity as well as e-commerce services. As for the “platform architecture & organizing model” perspective they offer the widest range of interfaces, use the most modern and advanced data analytics and security technologies and are also innovators in data governance developing it past a mere give-and-receive scheme, which seems to be necessary for their more advanced service offer. Interestingly enough, governmental actors are rather strongly involved, as they are the sole operators in half of those CS. The advanced port eco-systems also seem to be more open platforms, as they are mostly participant governed and include more external developers than CS from other groups. Finally, half of the innovation-oriented port-eco-systems work on a not-for-profit basis.

We labeled the second archetype group as “*B2B-focused CS*”. This is the smallest group as only six CS were allocated to it. They are clearly distinguishable from the other groups, as they solely focus on B2B markets and consequently, governmental actors are only external stakeholders. Furthermore, these CS are much more localized than the other groups and many of the ACS in our sample were allocated here (e.g., London Heathrow, Dallas Fort Worth Airport). While all of the B2B-focused CS offer coordinative communication services and logistical value-added services, not one offers extended services. All B2B-focused CS are working with a closed platform and governance model, as they are solely lead-organization governed and do not work with complementors at all. Most are based on pre-existing platforms and are run on a for-profit only basis. Finally, the B2B-focused CS are the group with the lowest information availability regarding the value capture perspective.

The third archetype group was labeled “*Non-profit CS*”, as they are clearly distinguishable from the other groups based on their business objective. Consequently, this group relies mostly on subsidies and the governmental actors play a very important role in their governance, as the majority is at least partially controlled by a governmental agency. They operate on a mostly local and sometimes national level and address along with B2G different B2B markets, from limited to extended stakeholder groups evenly. Many do not offer any extended services and the majority also does not offer any data analytics through the platform. Lastly, these CS are rather closed-off platforms, that, if at all, only allow selected partners to contribute functionalities. The only inland terminal CS was allocated to the Non-profit CS group.

We named the last archetype group “Non-specialized Single Windows”, as this is the largest group of CS, and they have the highest focus on a regional B2B-core and B2G market, thereby acting as a single window for core port stakeholders. Those CS focus on regulatory and coordinative communication services as well as logistical and port-related value-added services but include significantly less data value-added services. Some members of these groups do include extended services and use standard interfaces, viz., web interface and EDI/API. They mostly use local statistical data analytics and standard security features. Governmental actors are mostly involved stakeholders but do not operate the platform. Consequently, non-specialized Single Windows are mostly funded by fees and not subsidies. Like the Non-profit group, they are rather closed-off platforms but are run solely on a for-profit base.

### Detailed results

To objectivize the dimensions that best distinguish the four groups, we perform additional statistical analyses based on contingency tables with the clustering allocation as one of the two vectors and each of our taxonomy’s dimensions as the other (Appendix Table 5). First, we calculate Pearson’s chi-squared test of independence (Delucchi, 1983). Due to relatively small values in some contingency tables, we decided to also calculate the according p- values of Fisher’s exact test (cf. McCrum-Gardner, 2008). Lastly, to get an idea of the dependency strength between all dimensions, we calculated both Cramér’s V (Cramér, 1946) and a corrected Cramér’s V (Bergsma, 2013), which aims to overcome a bias for finite samples. Additionally, to identify in which dimensions each group is distinct, we tested all four identified groups individually for (in)dependence of the dimensions of the taxonomy, i.e., we tested the CS that were allocated to the one group against the CS from the remaining groups for each dimension, thereby dichotomizing the clustering allocation vector of the respective contingency table (for an example of the described contingency tables see Appendix Tables 8 and 9). The last column of Table 4, which presents the statistical results described above, shows which of the identified dimensions are distinctive for one or a set of archetype groups. Here, one can see that ten dimensions are distinctive for the “*Innovation oriented port eco-systems*”, but only six are distinctive for the “*B2B-focused CS*” and the “*Non-specialized Single Windows*” respectively. In future research, these results could be used to define a minimum set of dimensions that should be investigated depending on the archetype group(s) of CS’ involved in the study. For example, if two CS from the “*Innovation oriented port eco-systems*” and the “*B2B-focused CS*” archetype groups shall be compared, one should investigate dimensions such as the “Geographic scope” or “Communication services” as those are distinctive for both archetype groups.

**Table 4:** Contingency table analysis results for cluster analysis with significance level α = 0.1; in the last column (Clusters with a significant difference), the individual numbers represent clusters (archetype groups): 1- Innovation oriented port eco- systems, 2- B2B focused CS, 3- Non-profit CS, 4- Non-specialized Single Windows

Contigency table dimensions	Chi- Sq^+^	p-Value Chi-Sq^+^	Cramer's V^+^	Corrected Cramer's V^+^	p-Value (Fisher exact text)^+^	Clusters with significant difference ^++^
Clustering ↔ Market	78,4	0 ***	0,77	0,57	0 ***	1,2,3,4
Clustering ↔ Geographic Scope	22,3	0,001 **	0,50	0,36	0,002 **	1,2
Clustering ↔ Modes of transport	30,9	0,158	0,48	0,24	0,133	
Clustering ↔ Communication services	51,5	0 ***	0,62	0.50	0 ***	1,2
Clustering ↔ Value-added services	27,6	0,153	0,46	0,19	0,031 *	1,4
Clustering ↔ Extended services	25,1	0,123	0,44	0,20	0,139	1
Clustering ↔ Platform origin	14,1	0,003 **	0,57	0,36	0,006 **	2
Clustering ↔ Interface	23,6	0,169	0,42	0,18	0,142	3,4
Clustering ↔ Data analytics	30,6	0,002 **	0,48	0,33	0 ***	1,3,4
Clustering ↔ Data security	20,2	0 ***	0,68	0,44	0 ***	1,3
Clustering ↔ Data governance	4,6	0,203	0,32	0,13	0,301	1
Clustering ↔ Role of governmental actors	45,1	0 ***	0,58	0,46	0 ***	2,3,4
Clustering ↔ Decisional openness	6,8	0,078 *	0,39	0,21	0,082 *	1
Clustering ↔ Complementor openness	20,5	0,015 *	0,39	0,26	0,022 *	1
Clustering ↔ Business objective of operate	35,6	0 ***	0,90	0,61	0 ***	2,3,4
Clustering ↔ Funding	20,6	0,006 **	0,48	0,34	0,006 **	3,4
Clustering ↔ Funding continuity	3,8	0,702	0,21	0,00	0,756	
Clustering ↔ Payment model	13,8	0,191	0,32	0,17	0,209	

^+^- Based on average for IMP data

^++^- Based on seperate Fisher exact test evaluation of each individual cluster against the CSs in the remaining clusters

First, we present those three dimensions that relate to key aspects of CS (see Table 1), viz. “Market”, “Role of governmental actors”, and “Geographic Scope”. Additionally, we look at the “Extended services”, “Data governance”, “Decisional openness”, and “Complementor openness” dimensions as they differ significantly between the “*Innovation-oriented port eco-system”* archetype group and the remaining CS as well as the “Platform origin” dimension as it differs significantly between the “*B2B-focused CS*” archetype group and the CS not part of this archetype group. We focus on those five dimensions as they differentiate only one archetype group from the remaining CS (only one digit in the last column of Appendix Table 5), therefore implicitly indicating that the remaining CS are rather homogenous in the respective dimension. We do so despite some of the chosen dimensions not having significant p-values for the test of independence between the overall cluster allocation and the respective dimension (e.g., the “Extended services” dimension, which is significantly different between the “Innovation-oriented port eco-system” archetype group and the remaining CS, but not between all archetype groups), as these dimensions are clearly distinguishing singular archetype groups.

For the “Market” dimension, we find that it significantly differentiates all identified CS archetype groups individually but also collectively. Further, we find that the “*Innovation-oriented port eco-system”* archetype group addresses the broadest set of stakeholders, while the “*B2B-focused CS*” addresses the narrowest. The “Non-specialized Single Windows” archetype group focuses its B2B activities the most on the core group of port stakeholders. All CS but the ones from the “*B2B-focused CS*” archetype group also include B2G activities and only the CS from the “*Innovation-oriented port eco-system”* and the “*Non-profit CS”* archetype groups also include G2G activities, i.e., connecting multiple governmental stakeholders, thereby facilitating the communication and cooperation of governmental agencies.

The “Role of governmental actors” differentiates the identified archetype groups well also. The “*B2B-focused CS*”, for example, do not have a direct involvement of governmental actors, which means that they are affected by the regulation and legislation of governmental actors. Yet, they are not involved in the operation of the platforms. On the other hand, the “*Non-profit CS*” are highly influenced by governmental stakeholders, coupled with a need for subsidies for the funding of the platform and its operations. For the “*Non-specialized Single Windows*” archetype group, governmental stakeholders are commonly involved in the operation of the CS but are often only one of many involved stakeholders. Here, we assume that the governmental stakeholders, when involved in one way or the other, have a relatively strong influence on the mission and vision of CS with the effects on platform origin and service offer described below, as they seem to shift the focus away from short-term goals towards a longer-term perspective.

The “Geographic Scope” dimension shows a clear differentiation between some of the archetype groups. Especially the “*B2B-focused CS*” concentrate more on their local markets, i.e., the immediate port location they are applied to. Only some have a regional reach, meaning that they also cover the hinterland of the respective port. On the other hand, both the “*Non-profit CS*” and “*Non-specialized Single Windows*” archetype groups have a focus on regional markets and the “*Innovation oriented port eco-systems*” archetype group expanded its reach to a national level. In this context, national peculiarities, especially in the regulatory and legislative sphere, seem to play an important role as they are relevant for the service offer of CS. Even for the most basic services, i.e., “Communication services”, CS need to, on the one hand, comply with a multitude of laws and regulations, such as data protection laws and more, themselves, but on the other hand also guarantee compliance for participating stakeholders, which is critical in B2B and B2G environments with sensitive data. Accordingly, none of the CS has expanded internationally yet but rather realized an internationalization through extended services by connecting to other local (national) platforms.

With the “Extended services” dimension, we have to first distinguish two types of platform cooperation models. First, an example of what we would call a “platform of platforms” is an app store, which is a multi-sided innovation platform (Cusumano et al., 2019), that gives its users access to other platforms such as messaging services, social media platforms or ride-hailing platforms. An app store does not commonly give its users access to another app store, though, which would be what we call a “network of platforms”, i.e., platforms that connect horizontally, not vertically. For example, CS that implement inter-CS connectivity thereby create a network of connected local platforms as they offer their users more or less direct access to another platform with similar services. While the CS of the “*Innovation oriented port eco-systems*” archetype group are moving in the direction of such a network of platforms by integrating their information flows with each other on a supra-national level (IPCSA, 2020), they also seem to be more proactive in transforming into a platform of platforms locally, by integrating with external platforms such as last-mile delivery organization platforms or freight capacity booking platforms (e.g., INTTRA) (Elbert & Tessmann, 2021). As can be seen from Table 3, all CS that do not belong to the “*Innovation oriented port eco-systems*” archetype group make significantly less use of the extended services, with the “*B2B-focused CS*” not using them at all.

For the “Platform origin” dimension, the “*B2B-focused CS*” archetype group sticks out compared to the other CS which are still mainly developed from scratch, individually tuned for the respective port. New approaches are chosen for those “*B2B-focused CS*”, as they are mostly either based on pre-existing CS platforms or built based on a re-usable core platform, which is easily adaptable to changing circumstances. As an example, take Belgium- based Nallian (Nallian, 2021e), a provider of platform solutions, who developed both Brussel airport’s BRUCloud and with NxtPort a part of Port of Antwerp’s CS infrastructure. They then leveraged their basic platform to other airports around the world, such as Dalles Forth Worth airport’s DFWCC or London Heathrow’s HCC CS. We find that the likelihood of utilizing a re-used platform core is closely linked to the mission and vision of the respective CS providers. While those two aspects are not part of our business model taxonomy, as they heavily influence the decisions on business model aspects and lie therefore somewhat before or above the business model itself, we want to introduce them here briefly. The mission of a CS provider shall be the overarching set of goals that is supposed to be achieved, while the vision is an idea of how these goals are going to be achieved on the one hand and how the goals might be further developed in the future. We find that the CS of the “*B2B-focused CS*” archetype group has a higher focus on short-term goals in their mission and vision such as specific membership goals or breaking even within a set timeframe. Especially the latter goal leads to a certain cost pressure, which seems to result in more cost-conscious solutions, i.e., using (pre-)existing platforms for the setup of a CS, thereby saving both time and money, even if that means that the breadth of functionalities offered is smaller (“*B2B-focused CS*” do not offer any extended services for example). To be clear, we do not find any functionality-related restrictions stemming from the re-use of existing platforms. Still, we assume that the differences between the archetype groups origin from a difference in their mission and vision, as the “*B2B-focused CS*” only offer those functions that are actively demanded by their business customers, such as coordination services and value-added logistical services, but therefore are as cost-efficient as possible to not be seen as a “burden” by their customers. Additionally, they need to be able to make a profit, as they are the only archetype group that solely consists of for-profit operators and where governmental stakeholders have no direct involvement, i.e., do not support the funding.

Overall, the “Data governance” dimension cannot be used to distinguish the archetype groups (yet), but it is significantly different for the “*Innovation oriented port eco-systems*” archetype group compared to the remaining CS. The availability of different forms of data is highly relevant in all data-driven businesses (cf. Hartmann et al., 2016). Our data governance dimension can offer a new perspective on how to incentivize participants to share data on a B2B platform or, more generally, customers to share data with companies past a mere give-and-receive scheme, which is the basis of many (consumer-oriented) platform business models such as Google services (e.g., Li & Hecht, 2021; O’reilly, 2007) where users provide their data (e.g., location data) to receive free services based on data from other users (e.g., Google Maps). This is particularly relevant in the context of competitive B2B networks, as incentivization strategies from consumer-oriented platforms cannot be easily transferred to their B2B equivalents (Loux et al., 2020; Wallbach et al., 2019). Monetary compensation for providing data can raise the awareness of the value of such data and incentivize companies to share it, especially if they can decide at which price they want to share certain data. In this case, data is not worth what the platform operator decides it to be worth by offering a set of “free” services to the sharing member but can be individually determined per data type, which offers the possibility to also share highly valued data. We find that these data governance deviations are a rather new phenomenon, as only the Port of Antwerp’s CS introduced their new approach that users pay three different categories of fees, namely a monthly base fee and a transaction-based fee for the usage of the CS platform and a data fee which is set by the data owner for using this valuable data (Lievens, 2017). Accordingly, one cannot see a more considerable difference in the data governance row between different archetype groups of Table 3.

Lastly, the openness of the platform to complementors and who can be involved in decision making differs for the “*Innovation oriented port eco-systems*” archetype group compared to the remaining CS. It can also be used to distinguish all archetype groups from one another. Interestingly, the “*Innovation oriented port eco-systems*” archetype group is comparatively open to participants making decisions, even if they are not one of the leading organizations of the respective port. They are also much more open to complementors supplying the platform eco-system with their own services and platforms. We find that this is one of the reasons why these CS have a broader set of functionalities, especially extended services, available on their platforms. On the one hand, more services can be offered if more complementors are involved in the platform, as the development is distributed between many different organizations. On the other hand, these complementors are more motivated to contribute to the respective CS if they feel that they can influence the future of the platform by being involved in landmark decisions.

## Discussion

We developed a taxonomy with a total of four perspectives and eighteen dimensions. A Cluster analysis was used to show the applicability of the taxonomy and to identify archetypes of CS business models. As can be seen from the results in Table 3 in conjunction with Table 4, the distinction of the four archetype groups is not limited to a single perspective or dimension, which shows the necessity of the various dimensions to differentiate CS from one another.

To further investigate RQ1, we now want to compare our taxonomy to existing taxonomies, especially those listed in Table 1. First, the four perspectives of our taxonomy were derived from existing platform and digital business model taxonomies and classifications, as described in the taxonomy development section. While the detailed dimensions and even more so characteristics differ, some previous taxonomies cover all four perspectives that we utilized for our taxonomy, especially Weking et al. (2020) and Abendroth et al. (2021).

Both publications do not cover two of the key aspects of CS, viz. the influence of governmental stakeholders and the platforms’ local boundedness, though. The details addressed under the overarching perspectives differ strongly also. As shown above (Table 1), other platform or digital business model taxonomies do not cover all four perspectives of the taxonomy developed in this paper and accordingly focus on different aspects, being either too specific or too general for our context to be practical.

A more detailed view on the dimensions and characteristics of the four perspectives unveils the novel aspects of our taxonomy of CS business models compared to existing taxonomies (Table 1). We start by discussing the three key aspects of CS as identified in the domain background, viz. B2B focus, governmental influence and local boundedness and proceed with further dimensions that entail novel insights compared to extant literature.

As we generally found that only a minority of previous platform and digital business model taxonomies focused on B2B markets, our taxonomy adds some interesting insights. The market characteristics had to be differentiated into three different B2B stakeholder groups, from limited over core to extended, to account for differences in CS business models. This can also be relevant for other MSP business models, as platforms commonly focus on specific core stakeholders at first and expand their reach over various stages (Tan et al., 2015). A more detailed view on different B2B relationship groups can be helpful to better understand the development paths of MSPs and their business models in the future. Also, the market aspects that involve governmental actors (B2G & G2G) have not been part of previous platform or digital business model taxonomies. Only Passlick et al. (2021) consider “state” actors in their “Clients” dimension, which solely entails governmental entities such as the military purchasing services or products instead of private businesses. Our taxonomy goes a step further and considers governmental stakeholders as part of the platform eco-system. This means that governmental stakeholders can equivalently to business stakeholders interact with businesses (B2G) or with other governmental stakeholders (G2G) in various roles, e.g., as the platform operator, a data provider, a data or service consumer, a contributor to the service offer and more.

Accordingly, our taxonomy distinguishes which role governmental stakeholders take as part of the “Platform architecture & organizing model” perspective. To the best of the authors’ knowledge, this dimension has not been included in any platform or digital business model taxonomy before. We ascribe this to the fact that governmental involvement within the same type of platform commonly does not cover the same breadth as it does with CS. For example, research on e-government applications (e.g., Ebrahim & Irani, 2005) naturally has to focus on full governmental involvement, as it is by definition government-centric. Broader research topics that regularly involve governmental actors such as Public Private Partnerships (PPP) either compare a wide range of such arrangements with strongly varying contexts which hinders effective comparability (e.g., Susha et al., 2017) or do not investigate digital platform business models (e.g., Klijn & Teisman, 2003; Tang et al., 2010). Reducing governmental stakeholders to potential buyers of products or services (Passlick et al., 2021) or just not considering their potential involvement at all falls short in the context of MSPs. As we can see from the CS cases, even as an external stakeholder, a governmental actor can have a significant regulatory influence and therefore change the boundary conditions for the development path of MSPs and should accordingly be considered in the respective business model. When governments are deeply involved or even the sole operator, such as in the cases of Port of Rotterdam/Amsterdam’s Portbase CS or Port of Antwerp’s CS, a longer-term, innovation-oriented focus of the platform can be the result as they can influence the mission and vision of the platform. A deeper involvement also increases the government’s awareness of barriers and facilitators of the platform, which can lead to interventions, such as an adapted regulatory and legislative strategy or the availability of (additional) state funding.

Deep involvement of governmental stakeholders also implies a certain local boundedness of CS, which differentiates them from many other MSPs, as these governmental stakeholders are bounded to their respective area of jurisdiction themselves. For example, governmental actors from the Netherlands, which operate the Port of Rotterdam/Amsterdam’s Portbase CS cannot easily operate a similar platform in another country, as they have no legislative power there. While existing platform or digital business model taxonomies sometimes consider the geographic scope (Täuscher & Laudien, 2018) or the geographic distribution (Abendroth et al., 2021), none of the study objects is geographically bounded. Accordingly, the taxonomies cannot consider the effects that such boundedness can imply. As CS are maximally used on a national level, but not internationally so far, some of the recent developments that are likely based in this local boundedness can be interesting for MSP business model research, as some CS aim for an internationalization nonetheless.

As discussed in the detailed results section above, this internationalization of the platforms can then, despite the regulatory barriers, be realized through cooperation between platforms, i.e., what we call a network of (local) platforms, which is part of the “Extended services” dimension of our taxonomy. Apart from singular, theoretical papers (Baron & Mathieu, 2013), these advanced, platform-integrating services, their adoption barriers and effects on the platform participants have not been studied in detail yet. Singapore’s Networked Trade Platform or India’s PCS 1x, for example, offer a wide variety of extended services (Elbert & Tessmann, 2021) but have not been studied in detail within the past fifteen years. So far, this kind of service has also not been included in any platform or digital business model taxonomy. We assume this to be the case due to the lack of consumer-oriented MSP examples that follow such strategies. Microsoft recently announced a similar approach to expanding their Windows 11 app store offer by adding direct access to Amazon’s Android app store (Warren, 2021). Based on this, we speculate that the identified “network of platforms” concept might increase in importance for MSP business models in the future, despite it being a novel concept for related business model taxonomies.

Lastly, to the best of the authors’ knowledge, data security and data governance have not yet been included as dedicated dimensions in any digital or platform business model taxonomy. While data security is not a direct value offer of any CS, as found by Passlick et al. (2021) for some predictive maintenance business models, lacking data security or the feeling thereof has been identified as an important barrier to the implementation of CS (e.g., Posti et al., 2011; Rodon et al., 2008) as well as other digital innovations (e.g., Chen & Zhao, 2012; Lu et al., 2005; Saeed et al., 2012). Its integration into MSP business model taxonomies seems therefore necessary, given also that our cluster analysis showed significant differences in this dimension between the “*innovation-oriented port eco-system*” archetype group and the other CS in our sample.

The “Data governance” dimension is particularly relevant for the CS context (e.g., Chandra & van Hillegersberg, 2018), as a government can enforce the usage of a CS by regulation and legislation, but it cannot force companies to share highly sensitive data without facing major resistance from these companies (Carlan et al., 2016; Rodon et al., 2008). As companies in any context are hesitant to share critical and sensitive data with competitors, complementors or even “neutral” entities such as governmental actors, the “Data governance” dimension has implications for other MSP business models also: It presents a new incentivization scheme for sharing such data, especially in a B2B platform context. While some existing platform business model taxonomies have dimensions that point in a similar direction, none exhibits the same focus on data providers or owners. For example, Täuscher & Laudien (2018) distinguish key revenue stream, pricing mechanism, price discrimination and revenue source as the dimensions of the value capture perspective, which are all focused on the operator of a marketplace platform. Additionally, they include a price discovery dimension as part of the value creation perspective where they distinguish whether prices are fixed, set by sellers or buyers, set through auction or negotiation, which is then focused on the price for the customer buying a product or service from the platform and accordingly does not consider how participants of a platform can be incentivized to share data, which might be necessary for certain products and services. Abendroth et al. (2021) go one step further and include a participation incentives dimension as part of their actor ecosystem perspective. They distinguish whether the platform operator incentivizes the participation on a B2B co-creation platform through non-monetary means, through both non-monetary and monetary means or not at all. While Abendroth et al. (2021) add a view on a third group, besides the platform operator and the consumers of products or services of the platform, viz. the complementors and how they can be motivated to join the platform, they also do not include a dimension that explains how owners of critical data can be incentivized to share such data. Accordingly, our “Data governance” dimension adds this novel perspective on data sharing incentivization.

Some of the identified dimensions and some of the respective characteristics show more general applicability as they were transferred from existing taxonomies to the CS context. Those dimensions stem especially from the “Platform architecture & organizing model” and the “Value capture” perspectives. The “Decisional openness” and “Complementor openness” dimensions have been used for the taxonomy for B2B co-creation platforms with the same characteristics before (Abendroth et al., 2021). Also, the “Funding”, “Funding continuity”, and “Payment model” dimensions have been used similarly before (Täuscher & Laudien, 2018; Weking et al., 2020).

Many of the context-specific dimensions of existing taxonomies are not applicable to the CS context, such as the “Core value proposition” dimension of Abendroth et al. (2021) or the “Key activities” dimension of Passlick et al. (2021), which determine the focus of the respective company’s activities for the chosen focus (B2B co-creation (Abendroth et al., 2021); Predictive Maintenance (Passlick et al., 2021)). Others, such as the “Options for extensibility” dimension of Abendroth et al. (2021), would generally be applicable to the CS context but have not been found crucial enough to be included in our business model taxonomy. Interestingly, potential services from existing platform business model taxonomies can be identified that have not been applied to the CS context yet. Täuscher & Laudien (2018) identify review systems, i.e., the user or marketplace reviews on other participants, as one of the four value creation dimensions of marketplace business models, but no CS utilizes such functionalities yet. As our taxonomy is easily extendable, this could be added once adopted by the first CS.

## Conclusion

In this study, we presented a taxonomy for the classification of multi-sided platforms in competitive B2B networks with varying governmental influence, specifically for Port and Cargo Community Systems. To develop the taxonomy, we followed the suggestions of Nickerson et al. (2013) and examined a data set of 44 CS for which sufficient data was publicly available. Based on the taxonomy and a mapping of our data, we analyzed the business models of CS. On top of commonly used dimensions of platform and digital business model frameworks and taxonomies (e.g., target market) which were adjusted to the context, we also identified new dimensions, viz. three service dimensions of CS, data security and data governance as well as the involvement level of governmental actors. Using cluster analysis, we examined which archetypes of CS business models currently exist and identified four archetypes. We used a multiple-imputation method to overcome issues resulting from missing data which is a common problem in framework and taxonomy applications especially in the value capture dimension (e.g., Gimpel et al., 2018). This procedure can be used in other studies and contribute to an effective analysis. Our analysis of the archetypes showed that they differ significantly in many of the identified dimensions. We identified extended CS services, such as a horizontal integration of similar platforms (“network of platforms”) and e-commerce services, which have not yet been covered by extant literature (Moros-Daza et al., 2020), but have recently been implemented in various CS especially by what we labeled as “*Innovation-oriented port eco-systems”*. On the other hand, the comparison with platform taxonomies showed that a common functionality of consumer-oriented digital platforms has not been used on CS, viz. a review system. Data security and data governance approaches as crucial aspects of the CS business models have also been identified which can play an important role in creating trust amongst the diverse set of stakeholders involved in these MSP which have not yet been included in any MSP related business model taxonomy, though. Apart from the “*B2B-focused CS*”, all other CS also actively address B2G and sometimes even G2G markets. Consequently, we found governmental actor involvement in all CS, varying from the role of an external stakeholder to being the sole operator of the platform. Thus, especially the “*Non-profit CS*” rely heavily on subsidies for their funding, which also differentiates them from other platform and digital business models. The insights provided by this study increase our understanding of platform business models in the specific context of competitive B2B networks with varying governmental influence, both in theory and practice.

As with every research project, our study faces some limitations. First, while we tried to collect and use an exhaustive sample of CS for our research, we cannot rule out that more CS exist that are not included in our preliminary sample of 77 CS. We tried to address this issue by building on a previously compiled list from Moros-Daza et al. (2020) and extending it by an extensive Google and Google Scholar search. The latter is highly reliant on the search terms we used, viz. “Community System” and “Single Window”. If a CS-like platform is not labeled with one of these terms, it cannot have been included in our sample. Second, we only included those 44 CS into our final sample for which we were able to retrieve sufficient information. More information might be available from other sources that we did not use. Third, when mapping and clustering our sample with the developed taxonomy, we could not retrieve information on all dimensions. To address this data related uncertainty, we used a multiple imputation approach and statistical clustering methods that are considered to perform well under such conditions.

Despite these limitations, our study contributes to both academia and practice. It adds to the descriptive knowledge on multi-sided platform business models, as it develops a context-specific taxonomy for CS. It thereby answers the call of Moros-Daza et al. (2020) to add to the limited body of holistic CS research studies. The developed taxonomy allows to describe CS as well as their context in a structured way for future contributions in the field and therefor acts as a “*theory for analyzing”* according to Gregor (2006). This can be the basis and foundation of future contributions to develop more advanced theories which explain, predict or give design and action advice (de Reuver et al., 2018; Gregor, 2006). The taxonomy provides practitioners with a differentiated view on the configuration (options) of CS business models while the presented archetypes provide an aggregated view of CS business models. This can support both researchers and practitioners, in allocating existing CS in relation to peers but also to other digital platforms based on their characteristics which can ultimately support informed decision-making on CS and other platform developments (de Reuver et al., 2018). With our results we also lay the basis for future research to answer the calls of de Reuver et al. (2018) and Robey et al. (2008) for a deeper understanding of sectoral and geographic differences in the assimilation and factors affecting the success of digital platforms and inter-organizational systems respectively, thereby creating a better contextualization.

Multiple topics for further research can be identified based on the taxonomy on the one hand and the cluster analysis on the other hand. Based on the CS-specific taxonomy, we can identify at least five promising future research paths for CS research and general MSP research. First, the effects of geographic boundaries on MSP business models could be investigated, as we find that the local boundedness of CS might be one of the reasons for new and innovative ways of cooperation between different platforms. Second, regarding these new ways of cooperation, it would be interesting to find out more about the drivers and barriers of MSP cooperation, especially on a horizontal (“network of platforms”) but also on a vertical (“platform of platforms”) level, i.e., what drives the strategic decision of a MSP to cooperate with another MSP. The horizontal cooperation (“network of platforms”) is, to the best of the authors’ knowledge, a rather new phenomenon in the digital platform sphere, given that Microsoft only recently announced its plan to give its Microsoft app store users access to Amazon’s app store (Warren, 2021). Third, a deeper study of the effects of governmental involvement and influence on MSP business models would be helpful. For the CS context, we find that the most innovative CS have a comparatively deep involvement of governmental stakeholders. It would be interesting to find out if that is transferable to other MSP and, if yes, under which conditions or if it is just a coincidence. Governmental involvement might become more relevant for other platforms in the near future, given the recent uptake in regulation efforts on digital platforms (Feld, 2019; TaylorWessing, 2020). Fourth, one could study the effects of focusing on different scopes of B2B stakeholders on B2B MSP’s business model trajectories. First insights (Rodon et al., 2008) seem to point into the direction that the involvement of a too broad set of B2B stakeholders in an early stage can hinder the development of a MSP. It would be interesting to know what is the “right” group of B2B stakeholders to involve in which stage of the MSP lifespan and what this depends on. This can also be viewed in terms of complexity, as the MSP increases its complexity with a larger eco-system that it addresses. Lastly, it would be interesting to find out more about the effects of the new data governance arrangements that we find for CS in specific on MSPs and their business models in general. Multiple questions, such as “Are MSPs that utilize a monetary compensation scheme to incentivize the sharing of sensitive, critical and high-value business data more successful in collecting such data and how does this effect their business?” arise from this aspect.

Despite similar starting conditions, such as being locally bounded MSPs at port locations with some level of governmental influence, they develop a wide variety of business models, which can partially be based on the level of governmental influence but likely also on other local circumstances, such as the size and importance of the port for the respective country, the available infrastructure and others. Some CS are run solely on a for-profit basis, have no directly involved governmental stakeholders and accordingly focus on B2B stakeholders. They have a narrower functional scope than other CS, which are more innovation-oriented or focus on operating their platform as non-profit organizations. These different developments of MSP business models can be an interesting starting point for future research in the field, as different CS can be easily compared with the presented taxonomy but do not overlap due to the local boundedness aspect. For example, an in-depth longitudinal multiple case study of CS from different archetype groups could generate valuable insights. It could help to understand better which local factors lead to the different development paths in their business models. Additionally, future research could look into which dimensions are particularly relevant for a specific archetype group and the reasons behind it. Lastly, a CS specific future research direction could be to ask, why CS currently do not use any review systems/schemes despite them being used in many other MSP contexts.

Our statistical analysis of the clustering results provides initial explanatory approaches into which dimension of CS business models might influence each other. Appendix Table 10 which presents the statistical analysis performed on the mapping of our final sample, shows the dependencies between different dimensions. We see, for example, a medium strength dependency between decisional openness and extended service offer (χ2=13.4; p-value=0.037, Cramer’s V=0.55), which could be an explanatory approach to the wider range of services offered by “*Innovation-oriented port eco-systems*” compared to the other CS, as they rely much more on all of their participants for decision-making. Future research can extract further, similar hypotheses from our analysis and test them on both CS in specific but also MSP in general. Appendix Table 6 in conjunction with Appendix Table 7 also reveals three aspects that can be relevant for future research on CS in specific. First, we can see that in our data-rich samples A and B in Appendix Table 6, CS from the “*B2B-focused CS*” archetype group are highly underrepresented, which means that B2B-focused CS have received significantly less research attention, although they might face different adoption barriers and outcomes. Second, “*Innovation oriented port eco- systems*” such as Singapore’s Networked Trade Platform or India’s PCS 1x offer a wide variety of extended services (Elbert & Tessmann, 2021), but have not been studied in detail within the past fifteen years, so their recent developments have not been analyzed in detail. Lastly, when mapping CS against their respective continent, one can see that especially CS for African ports are underrepresented in peer-reviewed studies and that no Southern American CS had enough data available to be included in our sample.

Finally, it has to be noted that the taxonomy as well as the identified archetypes must be checked in the future as changes may occur. New technologies as well as new stakeholders, such as digital freight forwarders have the potential to significantly change the market situation which can cause the taxonomy to change over time. A revision of characteristics may be needed, and it may be necessary to consider other dimensions. Accordingly, we developed our taxonomy to be revisable and extendible as suggested by Nickerson et al. (2013).

## Figures and Tables

**Fig. 2:**
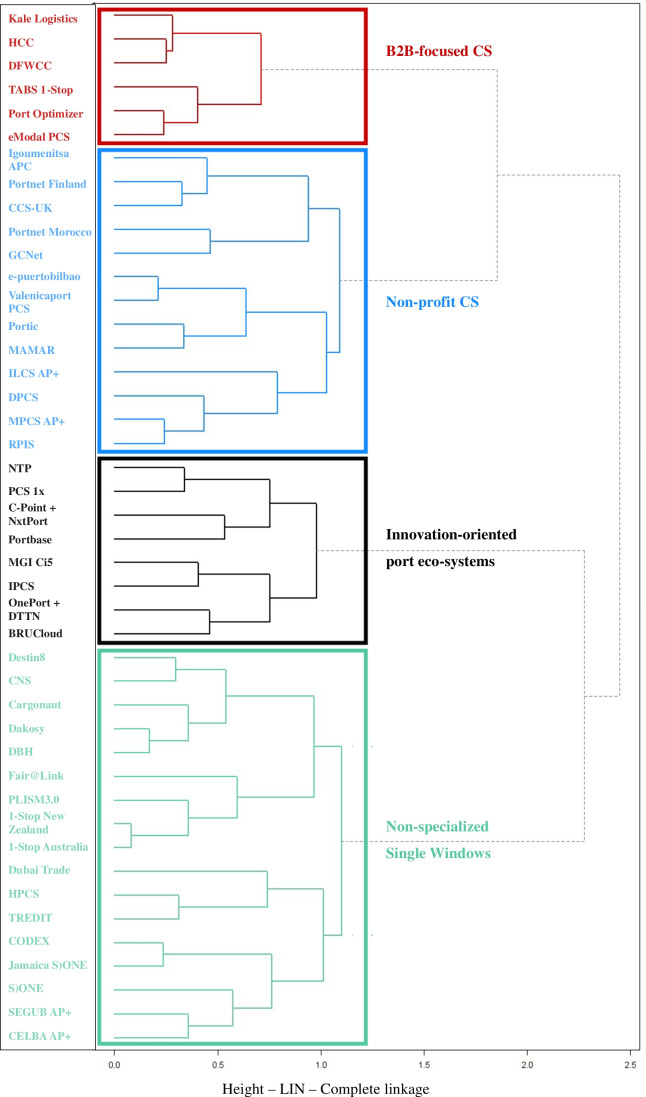
Dendrogram for hierarchical agglomerative clustering of WOM data with LIN distance and complete linkage method

**Fig. 3:**
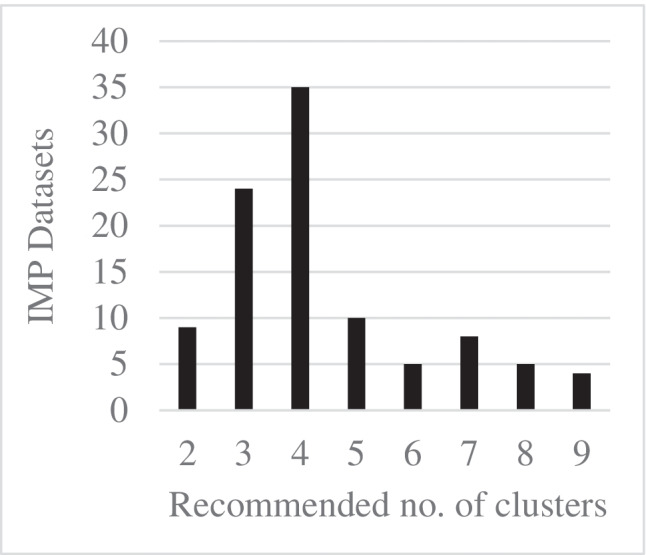
Distribution of recommended clusters according to the knee method based on 100 imputed datasets (IMP data)

**Table 7:**
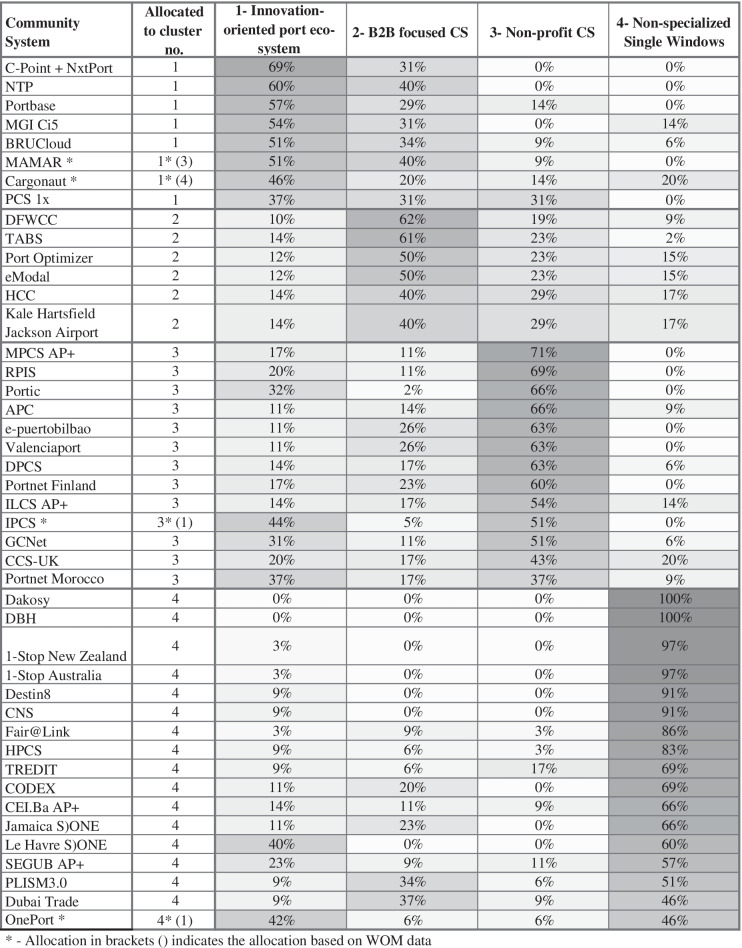
Cluster allocation distribution per Community System for those 35imputed datasets {IMP data) which suggested k=4 cluster.;. Based on hierarchical agglomerative clustering with LIN distance and complete linkage

## Appendix

### Platform and digital business model taxonomies semi-structured literature search

• *Selection of sources:* We followed the procedure described by Durach et al. (2017) for a semi-structured literature search on existing platform and digital business model taxonomies. As the aim of this paper is not a structured literature review on platform and digital business model taxonomies, we only utilized a search on Google Scholar as it is estimated to cover the broadest range of records of scientific literature (Gusenbauer, 2019) and is mainly viewed as an appropriate literature search tool despite shortcomings such as a lack of quality control (Halevi et al., 2017; Martín-Martín et al., 2021).

• *Utilized keywords:* We used a rather inclusive than exclusive keyword approach in line with the recommendations of Durach et al. (2017) to retrieve a baseline sample. Specifically, this means that we first searched for the terms “Platform taxonomy”, “Platform business model taxonomy”, and “Digital business model taxonomy”. In our search, we also replaced the term ”taxonomy” with “classification” and “framework”, as we found that these terms were used in proximity with taxonomies.

• *Inclusion/exclusion criteria:* We included only peer-reviewed journal papers and conference proceedings. We include literature in English language only, as it is the dominant research language. Lastly, we excluded any literature that does not investigate a taxonomy or classification framework with dedicated characteristics describing either a platform or digital business model. This was the focus of our literature search. Due to the fast changes in business models related to digital products and services, we only included results from the past five years, i.e., published from 2016 until 2021.

### Port and Cargo Community System (CS) data collection

• *Selection of sources:* Szopinski et al. (2020) recently used a search-engine based approach to identify relevant Business Model Development Tools for their respective taxonomy. We want to follow this approach and also utilize scholarly and non-scholarly search engines (i.e., Google Scholar and Google). Google Scholar is estimated to cover the widest range of records of scientific literature (Gusenbauer, 2019) and is mostly viewed as an appropriate literature search tool despite shortcomings such as a lack of quality control (Halevi et al., 2017; Martín-Martín et al., 2021). For both search engines we screened the first 1000 search results for identifying additional CS as we did not identify any new CS after the 800^th^ result and for the enrichment on information on each CS the first 350 search results, as we never found any relevant results from the 250^th^ search result onwards.

• *Utilized keywords:* We used a rather inclusive than exclusive keyword approach in line with the recommendations of Durach et al. (2017) to retrieve a baseline sample. Specifically, this means that we first searched for the terms “Community System” and “Single Window”, thereby also covering the terms “Port Community System”, “Cargo Community System” and “Airport Community System”. When searching for more information on a specific, already identified CS, we used the name of the respective CS (e.g., “Portic” for the PCS of the Port of Barcelona (cf. Rodon et al., 2008)) as the search term.

• *Inclusion/exclusion criteria:* We included all types of literature, including, but not limited to journal papers, conference proceedings, monographs, newspaper articles, company websites and practitioner-oriented blogs. We include literature in German and English language as those are the languages we are proficient in and because we wanted to cover an as wide sample as possible. Lastly, we excluded any literature that does not describe or investigate any characteristics of specific CS, i.e., theoretical papers on CS for example.

• *CS search procedure:* Similar to the procedure described by Szopinski et al. (2020), we ran the searches independently on the web browser’s incognito mode and additionally used various VPN services to change the virtual location to other regions (e.g., USA, Argentina, Columbia, South Africa, Thailand and others) to avoid corrupted search results from previous usage of the search engine as well as our physical location. In our first step we aimed to identify further CS on top of the 48 PCS found by Moros-Daza et al. (2020). Our preliminary sample included 72 CS (Appendix Table 6), i.e., we found 24 additional CS compared to Moros-Daza et al. (2020). If a CS is used in multiple locations (e.g., Community Network Services (CNS) in Great Britain (Moros-Daza et al., 2020)), we only considered the CS with the largest range of functionalities and users. During the second step of our search process on further information on the identified CS, we recognized that for some CS of our preliminary sample, we could not find any usable information regarding business model characteristics. Those were excluded from our preliminary sample subsequently to come to our final sample of 44 CS (Appendix Table 6) with at least one peer-reviewed source and one non-peer reviewed source or three non-peer reviewed sources with information on characteristics of the respective CS.

To present a brief overview of the data that we extracted from the sources named in Appendix Table 6 for the taxonomy development and the CS clustering, we want to briefly present some examples of the collected data. Take, for example, Chandra & van Hillegersberg (2018) who show in their 5^th^ figure a detailed breakdown of stakeholders involved in Port of Rotterdam’s and Amsterdam’s CS (Portbase). From their analysis, we can derive certain characteristics of dimensions of our taxonomy. For example, Chandra & van Hillegersberg (2018) show that governmental authorities, i.e., in Portbase’s case the port authorities of Rotterdam and Amsterdam, are the sole operators of the CS, but decisions are participant governed, as the CS’ boards involve non-governmental stakeholders from the member and other groups as well. Dimensions from the Value creation (What?) and Value Capture (Why?) perspectives were mostly extracted from CS operators’ websites as peer-reviewed sources rarely covered these aspects, with the exception of Carlan et al. (2016) for Port of Antwerp’s PCS, who indicate that APCS does not charge fees for information use and Elbert & Tessmann (2021) who compare the value creation perspective of four PCS. An example for a source that is neither peer-reviewed nor a port’s web page would be the publicly available presentation of Lievens (2017), CEO of NxtPort of Port of Antwerp, where he describes the data monetization rules of NxtPort on page 24, which is the basis for the data governance characteristic “Monetary compensation”.

### Taxonomy development

**Table 5:** Fulfilled ending conditions per iterations. Analogue to (Passlick et al., 2021), based on ending conditions of (Nickerson et al., 2013)

Iteration				Ending conditions according to Nickerson et al. (2013)
1. con.	2. emp.	3. emp.	4. emp.	
Subjective				
	X	X	X	Concise
	X	X	X	Robust
		X	X	Comprehensive
X	X	X	X	Extendible
		X	X	Explanatory
Objective				
			X	All objects have been examined
X	X	X	X	No object was merged with a similar object or split into multiple objects in the last iteration
	X	X	X	At least one object is classified under every characteristics of every dimension
			X	No new dimensions/characteristics were added in the last iteration
		X	X	No dimensions/characteristics were merged/split in the last iteration
X	X	X	X	Every dimension in unique and not repeated
X*	X*	X*	X*	Every characteristic is unique within its dimension
X	X	X	X	Each combination of characteristics is unique and not repeated

* For a more concise depiction of the characteristics, Nye deviate from (Nickerson et al., 2013) in that we do not use mutually exclusive characteristics for all dimensions in Table 1

**Table 6:** Community System samples with port name, Community System name, the source through which the CS was identified and utilized peer- and non-peer-reviewed sources. Data sources were used for both taxonomy development and CS clustering

Sample	Port Name	CS/Operator Name	Identification source	Content sources (peer-reviewed)	Content sources (other)
Sample A – CS with excellent information availability					
A	Port of Rotterdam and Amsterdam (Netherlands)	Portbase + Trade & Transport Gateway	(Moros- Daza et al., 2020)	7 (Chandra & van Hillegersberg, 2018; de Langen & Heij, 2014; de Langen, 2005; Simoni et al., 2020; Taneja et al., 2010; van der Horst & van der Lugt, 2011, 2014)	4 (Constante, 2019; Keretho & Pikart, 2013; Portbase, 2020; Van Baalen et al., 2009)
A	Port of Hamburg (Germany)	Dakosy	(Moros- Daza et al., 2020)	6 (Carlan et al., 2016; Fedi et al., 2019; Heilig & Voß, 2017; Kapkaeva et al., 2021; Matczak, 2013; Simushkov & Korovyakovsky, 2008)	7 (Constante, 2019; DAKOSY, 2020a, c; IPCSA, 2020; Keretho & Pikart, 2013; UNINA & RAM, 2015; Van Baalen et al., 2009)
A	Port of Singapore (Singapore)	Networked Trade Platform [NTP] (Portnet + Tradenet)	(Moros-Daza et al., 2020)	5 (Gordon et al., 2005; Lee-Partridge et al., 2000; Neo, 1994; Neo et al., 1994; Teo et al., 1997)	7 (DTledger, 2020; NTP, 2020; Sathasivam, 2009; Singapore Customs, 2018, 2020; The Business Times, 2018; Van Baalen et al., 2009)
A	Ports of Le Havre (France)	SOGET Le Havre PCS (S)ONE)	(Moros- Daza et al., 2020)	4 (Baron & Mathieu, 2013; Carlan et al., 2016; Fedi et al., 2019; Joszczuk– Januszewska, 2012)	7 (Bunker Ports News Worldwide, 2019; de Blic et al., 2018; FAQ Logistique, 2018; IPCSA, 2020; SOGET, 2019, 2020e; UNINA & RAM, 2015)
A	Thessaloniki Port (Greece)	TREDIT (FRETIS_IFT)	(Polydoropoul ou et al., 2011)	4 (Arduino et al., 2013; Giannopoulos, 2008, 2009; Polydoropoulou et al., 2011)	2 (TREDIT, 2014, 2020)
A	Port of Barcelona (Spain)	Portic	(Moros- Daza et al., 2020)	3 (Rodon et al., 2007, 2008; Rodon & Ramis- Pujol, 2006)	4 (Constante, 2019; IPCSA, 2020; Portic, 2020; Portic Barcelona, 2015)
A	Port of Valencia (Spain)	Valenciaport PCS	(Moros- Daza et al., 2020)	3 (Carlan et al., 2018; Furio, 2013; Vieira et al., 2014)	4 (Diaz, 2003; ValenciaportPCS, 2012, 2020a, b)
A	Ports of Felixstowe and others (UK)	MCP plc’s Destin8 Port Community System	(Moros- Daza et al., 2020)	3 (Hulstijn et al., 2016; Long, 2009; Rukanova et al., 2018)	4 (Constante, 2019; IPCSA, 2020; Keretho & Pikart, 2013; MCP, 2020)
A	Port of Antwerp (Belgium)	C-Point + NxtPort (former APCS)	(Moros- Daza et al., 2020)	2 (Carlan et al., 2016; Jović et al., 2019)	11 (Antwerp Port Authority, 2015; Carlan et al., 2018; Constante, 2019; IPCSA, 2020; Lievens, 2017; Moyersoen, 2019; NxtPort, 2018, 2020a, b; Port of Antwerp, 2020; Waterschoot, 2011)
A	Port of Hong Kong (Hong Kong)	OnePort + DTTN	(Cheng & Wang, 2016)	2 (Carlan et al., 2016; Cheng & Wang, 2016)	6 (DTTN, 2021; Hang Seng Bank, 2020; HIT, 2003; Hong Kong Shippers’ Council, 2005; OnePort, 2020; OnePort Limited, 2014)
A	Ghana ports (Ghana)	GCNet	(Devex, 2021)	2 (Adaba & Rusu, 2014; Osei-Owusu & Mahmood, 2020)	4 (Acquah-Bentil, 2015; Agyemang, 2016; Devex, 2021; Wulf, 2004)
A	Amsterdam Schiphol (Netherlands)	Cargonaut	(Moros- Daza et al., 2020)	2 (Chandra & van Hillegersberg, 2019; Gudmundsson & Walczuck, 1999)	3 (Cargonaut, 2021; Smith, 2019; Stat Times, 2020)
A	UK airports—various (UK)	CCS-UK	(CCS-UK, 2021)	2 (Damsgaard, 1999; Gudmundsson & Walczuck, 1999)	3 (CAAS, 2018; CCS- UK, 2021; DVZ, 2020)
Sample B – CS with good information availability					
B	Ports of Marseilles (France)	MGI Ci5 (former AP +)	(Moros- Daza et al., 2020)	1 (Baron & Mathieu, 2013)	8 (Constante, 2019; FAQ Logistique, 2018; Grandjean, 2019; ITJ, 2015; MGI, 2020a, b, c; UNECE, 2017)
B	Frankfurt Airport (Germany)	Fair@Link	(Wallbach et al., 2019)	1 (Wallbach et al., 2019)	4 (Brett, 2020; DAKOSY, 2020b; Gladiator, 2020; Woywod, 2015)
B	Ports of Morocco (Morocco)	Portnet	(Moros- Daza et al., 2020)	1 (Jouad & Hamri, 2020)	4 (Hafsi & Benhayoun, 2017; IPCSA, 2020; Keretho & Pikart, 2013; PORTNET, 2020)
B	Port of Igoumenitsa (Greece)	Igoumenitsa APC	(Tsamboulas & Ballis, 2013)	1 (Tsamboulas & Ballis, 2013)	3 (Grinias et al., 2015; PORT AUTHORITY OF IGOUMENITSA, 2014; Reuters, 2020)
B	Port of Los Angeles & Long Beach + 12 more ports (USA)	eModal PCS	(Moros- Daza et al., 2020)	1 (Huynh et al., 2016)	3 (Advent Intermodal Solutions, 2020; Hoang et al., 2014; Petrakakos, n.d.)
B	Ports of Korea (Korea)	KL Net PLISM3.0 (former Port- MIS)	(Moros- Daza et al., 2020)	1 (Lee et al., 2000)	3 (Google Play, 2021; KLNET, 2020; Young Park & Ik Yun, 2018)
B	Port of India (members of the Indian Ports Association) (India)	PCS 1x	(Moros- Daza et al., 2020)	0	10 (Constante, 2019; Indian Ports Association, 2020; IPCSA, 2020; J.M. Baxi Group, 2019; Maritime Gateway, 2020a, b, c; Mathew, 2019; Portall, 2020; Roychowdhury, 2019)
B	Abu Dhabi Ports (United Arab Emirates UAE)	Maqta Gateway (MAMAR[mPC S] + Margo)	(Moros- Daza et al., 2020)	0	7 (Abu Dhabi Ports, 2018; Desormeaux, 2016; IAPH, 2019; IPCSA, 2020; Kamel, 2020; KIZAD, 2020; Margo, 2020)
B	Port of Bilbao (Spain)	e-puertobilbao	(Moros- Daza et al., 2020)	0	6 (Apple App Store, 2020; Bilbaoport, 2020; e-puertobilbao, 2016, 2020; IPCSA, 2020; Marin Portillo & Basterrechea Iribar, 2020)
B	Port of Louis (Mauritius)	SOGET (MPCS AP +) + TradeNet	(Moros- Daza et al., 2020)	0	6 (Ancharaz, 2018; Essay UK, 2020; MACCS, 2017, 2020; Ng Cheong Hin, 2013; SOGET, 2020f)
B	Kingston port (Jamaica)	SOGET (Jamaica S)ONE PCS)	(Moros- Daza et al., 2020)	0	5 (Jackson, 2016; Linton, 2020; Powell, 2017; SOGET, 2020d; Wyciwski, 2016)
B	Port of Bremen (Germany)	BHT DBH	(Moros- Daza et al., 2020)	0	5 (dbh Logistics IT AG, 2011, 2020; Hedde-von Westernhagen & Sarigül, 2020; Jaklitsch, 2013; Keretho & Pikart, 2013)
B	Port of Jakarta (Indonesia)	ILCS AP + PCS	(Moros- Daza et al., 2020)	0	5 (Hutchinson Whampoa Limited, 2003; ILCS, 2018, 2020; Leach, 2012; SOGET, 2020c)
B	Port of Los Angeles & Long Beach (USA)	Wabtec Port Optimizer	(Moros- Daza et al., 2020)	0	5 (Mongelluzzo, 2018a, b; Port of Los Angeles, 2020; Wabtec Corp, 2020; WorldCargo News, 2018)
B	Port of Lyttelton (New Zealand)	1-Stop PCS	(Moros- Daza et al., 2020)	0	5 (1-Stop, 2019, 2020a, b; IPCSA, 2020; Port Strategy, 2017)
B	Ports of Australia (Australia)	1-Stop PCS	(Moros- Daza et al., 2020)	0	5 (1-Stop, 2019, 2020a, b; IPCSA, 2020; Port Strategy, 2017)
B	Ports of Israel (Haifa & Ashdod & Eliat) (Israel)	Israel Port Community System (IPCS)	(Moros- Daza et al., 2020)	0	5 (Ben-Moshe, 2017; Cyberark, 2020; IPCSA, 2020; Israel Port Authority, 2020; Israel Ports, 2020)
Sample C – CS with sufficient information availability					
C	Brussels Airport (Belgium)	BRUCloud	(Schoeters, 2016a)	0	4 (Barnett, 2018; Brussels Airport, 2021; Schoeters, 2016a, b; van Gelder, 2020)
C	Dubai Ports World (United Arab Emirates UAE)	Dubai Trade	(Moros- Daza et al., 2020)	0	4 (DP World, 2020; Dubai Trade, 2016; IPCSA, 2017; Trade Finance, 2012)
C	Guadeloupe ports (Guadeloupe)	SOGET AP + CEI.BA	(SOGET, 2021)	0	4 (CEI.BA, 2021; Foucault, 2019; SOGET, 2021; UNECE, 2017)
C	Hartsfield- Jackson Atlanta Airport (USA)	Kale Logistics	(Kale Logistics, 2019)	0	4 (Kale Logistics, 2019, 2020b; PayCargo, 2020; Putzger, 2020a)
C	Mulhouse, Colmar/Neuf- Brisach, Strasbourg (France), Basel (Switzerland), Weil a.R., Kehl, Karlsruhe, Mannheim, Ludwigshafen (Germany)	RheinPorts Information System (RPIS)	(de Blic et al., 2018)	0	4 (de Blic et al., 2018; DVZ, 2017; Interreg, 2019; Wiech, 2019)
C	Piraeus (Greece)	Hellenic PCS (HPCS)	(HPCS, 2021)	0	4 (Amditis, 2020; Bellos, 2020; Fanti et al., 2020; HPCS, 2021)
C	Port of Cotonou (Benin)	SEGUB AP + PCS	(Moros- Daza et al., 2020)	0	4 (Nathan Associates, 2013; PMAC, 2017; SOGET, 2011, 2020a; World Bank Group, 2015)
C	Ports of Finland (Finland)	Portnet Finland	(Moros- Daza et al., 2020)	0	4 (Arkima, 2017, 2019; Leviäkangas & Hautala, 2002; Tulli, 2020)
C	Dallas Fort Worth Airport (USA)	DFW Cargo Cloud (DFWCC)	(Putzger, 2020a)	0	3 (Blasquez, 2020; Nallian, 2021a; Putzger, 2020a)
C	London Heathrow Airport (UK)	Heathrow Cargo Cloud (HCC)	(CAAS, 2018)	0	3 (CAAS, 2018; Heathrow Airport, 2017; Nallian, 2021b)
C	Port of Djibouti (Djibouti)	Djibouti port community system (DPCS)	(Moros- Daza et al., 2020)	0	3 (Crimsonlogic, 2018; DPCS, 2020; IPCSA, 2020)
C	Port of Manila (Philippines)	TABS 1-Stop	(Moros- Daza et al., 2020)	0	3 (1-Stop, 2018; 1-Stop TABS, 2015; IPCSA, 2020)
C	Port of Tuticorin (India)	Kale Logistics (CODEX)	(Moros- Daza et al., 2020)	0	3 (Kale Logistics, 2020a; Pereira, 2020; Shaikh, 2018)
C	Ports of Southampton, Tilbury, London, Heathrow, Gatwick, Manchester, Birmingham, Immingham, Belfast and Portsmouth (UK)	Community Network Services (CNS)	(Moros- Daza et al., 2020)	0	3 (CNS, 2021; DP World Southampton, 2020; HSN, 2020)
CS with insufficient information availability					
-	DP World Yarimca (Turkey)	Dubai Trade	(Moros- Daza et al., 2020)		
-	Port of Tarragona, Port of Huelva, Port of Bahia de Algeciras, Port of Galicia (Spain)	Portel	(Moros- Daza et al., 2020)		1 (Keretho & Pikart, 2013)
-	Port of Shanghai (China)	SIPG PCS	(Moros- Daza et al., 2020)		
-	Ports of Dover, Portland, Poole and Scrabster (UK)	Pentant	(Moros- Daza et al., 2020)		
-	Port of Dalian (China)	Dalian Portnet	(Moros- Daza et al., 2020)		
-	Port of Genoa (Italy)	E-port (integrated with UIRNet Piattaforma Logistica Nazionale (PLN))	(Moros- Daza et al., 2020)		1 (PCS e-port, 2008)
-	Port of Ravenna (Italy)	Port of Ravenna PCS	(Moros- Daza et al., 2020)		1 (Autorita Portuale di Ravenna, 2020)
-	Port of Savonna (Italy)	PCS Savonna	(Port Authority of Savonna, 2021)		1 (Port Authority of Savonna, 2021)
-	Port of Sines (Portugal)	Port of Sines JUP	(Moros- Daza et al., 2020)		2 (de Sena Pedro Salvador, 2017; Ports of Sines and the Algarve Authority, S. A, 2020)
-	Port of Montréal (Canada)	Port of Montréal PCS	(Moros- Daza et al., 2020)		(De la Guia, 2013)
-	Port of Abidjan (Ivory Coast)	SOGET POS (AP +)	(Moros- Daza et al., 2020)		
-	Ports of Kinshasa, Boma, Goma, Kinsagani, Mati (Congo)	SOGET (DR of Congo Port Community System S)ONE)	(Moros- Daza et al., 2020)		(SOGET, 2020b)
-	Port of Odessa (Ukraine)	PPL 33–35	(Moros- Daza et al., 2020)		(IPCSA, 2020)
-	Ports of Togo (Togo)	SOGET (SEGUCE Togo AP +)	(Moros- Daza et al., 2020)		(SOGET, 2020g)
-	Ports of Trieste (Italy)	Ports of Trieste PCS Sinfomar	(Moros- Daza et al., 2020)		(IPCSA, 2020; Sinfomar, 2015)
-	Gdańsk, Gdynia and Szczecin- Świnoujście (Poland)	PolskiPCS—still under development	(Marek, 2017)		
-	Gothenburg port (Sweden)	Wabtec Port Optimizer	(Port Technology, 2020)		
-	Port of New York/New Jersey (USA)	eModal (PortTruckPass 2.0 – former TIPS)	(Port of New York and New Jersey, 2021)		
-	Port of Santos (Brazil)	JUP	(Moros- Daza et al., 2020)		
-	Sydney, Brisbane, Fremantle, Flinders (Australia)	CommercePlus (PortBIS)	(Gil-Campos, 2016)		
-	Port of Prince Rupert (Canada)	Port of Prince Rupert PCS	(Moros- Daza et al., 2020)		
-	Liege Airport (Belgium)	LGG Cargo Cloud (similar to BRUCloud)	(Nallian, 2021c)		
-	Luxembourg Airport (Luxembourg)	lux-airport	(Nallian, 2021d)		
-	Vienna International Airport (Austria)	VIE Cloud	(Nallian, 2021f)		
-	Port of Valparaiso (Chile)	SILOGPORT	(Vairetti et al., 2019)		
-	Port of Buenos Aires (Argentina)	e-PuertoBue	(Vairetti et al., 2019)		
-	Mumbai airport (India)	Kale Logistics	(Patwardhan, 2018)		
-	Port of Leghorn (Italy)	TPCS	(Di Vaio & Varriale, 2020)	1 (Di Vaio & Varriale, 2020)	
-	Ports of Levante (Italy)	GAIA	(Di Vaio & Varriale, 2020)	1 (Di Vaio & Varriale, 2020)	

### CS clustering

Our requirements are very similar to those proposed by Gimpel et al. (2018) and Passlick et al. (2021). Both publications utilize an agglomerative hierarchical clustering algorithm (Ward, 1963) to identify relevant clusters. A highly important step when utilizing this, and any other clustering algorithm, is to choose an appropriate distance or dissimilarity measure, i.e., to define how one measures the similarity of two objects (e.g., Finch, 2005). Both Gimpel et al. (2018) and Passlick et al. (2021) utilize the matching coefficient distance (Sokal & Michener, 1958). This distance measure is defined for binary variables only and considers two objects as close to each other, if many variables show co-occurrence (i.e., a variable is 1 for both objects) and/or co-absence (i.e., a variable is 0 for both objects) (cf. Batyrshin et al., 2016). For this distance measure, categorial data has to be dichotomized (Gimpel et al., 2018; Passlick et al., 2021). Dichotomization creates a high-dimensional problem as multiple variables are created to represent what was previously one (categorial) variable. This can be problematic if, as in our case, the sample size is relatively small (Jain et al., 2000; Peng et al., 2020; Wang et al., 2008). Therefore, we take a different approach, based on a similarity measure that was developed specifically for categorial data (Boriah et al., 2008). Šulc & Řezanková (2019) recently noted that Ward’s algorithm cannot be utilized for categorial data as the concept of centroids is not defined for this context. Instead, they recommend the *Lin* similarity measure combined with complete linking for hierarchical clustering of datasets with a higher number of variables (Šulc & Řezanková, 2019). “*The Lin measure gives higher weight to matches on frequent values, and lower weight to mismatches on infrequent values*.” (Boriah et al., 2008, p. 249), which seems to be a good fit for our context, as it is less susceptible to outliers in singular dimensions. To determine the appropriate number of clusters, we use the “knee” method, i.e., identifying the number of clusters where the merge distance curve has its maximum curvature, which is an accepted method for hierarchical clustering (Salvador & Chan, 2004).

Due to lacking information, we have some missing data for the “Funding”, “Funding continuity” and “Payment model” dimensions of our “Value capture” perspective. Initially, we performed the cluster analysis on all 44 CS excluding the dimensions with incomplete data and call those “WOM”-cluster results. The dendrogram in Appendix Figure 2 shows a clear distinction into four groups of the WOM results, which was the result of the “knee” analysis.

A better alternative to data omission is data imputation, i.e., filling in missing values with artificial values, either on a random basis, based on correlations of other variables or other statistical methods (Efron, 1994; Van Buuren, 2018). (Multiple) imputation is used in the context of clustering regularly (Choudhury & Kosorok, 2020) and is recommended even for variables with a high proportion of missing values (cf. Madley-Dowd et al., 2019). Multiple imputation (MI) is an approach where missing values are imputed *m* times, to create *m* different completed datasets which can be consecutively evaluated and aggregated in a final step (Lee & Simpson, 2014). We follow the framework for reporting on cluster analysis when using multiple imputation by (Basagaña et al., 2013). Firstly, the number of imputations have to be determined. Commonly, only 3 to 5 imputations are recommended (Schafer & Olsen, 1998), but newer research suggests, that especially in cases where the fraction of missing data is comparably high and if the dataset that is imputed is relatively small, much higher numbers are necessary (Carlin et al., 2003; Graham et al., 2007). As both is the case for our data, we follow (Graham et al., 2007)’s suggestion to use 100 imputations. Based on recent results from (Akande et al., 2017) on MI methods for categorical data, we choose the classification and regression trees (CART) imputation method, as it was found to be robust, deliver good results and is readily available in the software tool “R” through the “mice” package.

Accordingly, we created 100 imputed data sets (“IMP” results) and ran the “knee” method to identify the recommended number of clusters for each of the 100 data sets. The distribution of the IMP results for the “knee” method can be seen in Appendix Figure 3. Imputation has an effect on the number of recommended clusters although the most recommended cluster number of four clusters is the same as for the WOM case. Following the guidelines of Basagaña et al. (2013), we only included those 35 IMP datasets, for which four clusters were recommended, for the succeeding cluster allocation process.

The cluster allocation of singular CS varies over the IMP datasets (Appendix Table 7) which is an expected outcome (Basagaña et al., 2013). Some CS, such as the ones of Hamburg and Bremen, were always allocated to the same cluster. On the other hand, the Moroccan CS was allocated evenly between two clusters. In this specific case, we decided to allocate it to the cluster that it was allocated to by the WOM data. The clustering based on the IMP datasets changed the cluster allocations only slightly, as four CS were reallocated, viz. the CS of Israel (IPCS), Abu Dhabi (MAMAR), Hong Kong (OnePort + DTTN) and Amsterdam Schiphol (Cargonaut).

**Table 8:** Example of contingency table for the CS taxonomy’s dimension "Geographic scope"

		Geographic scope		
		Local	Regional	National
Cluster	1 - Innovation oriented port eco-systems	0	2	6
	2 - B2B focused CS	3	3	0
	3 - Non-profit CS	0	8	5
	4 - Non-specialized Single Windows	2	13	2

**Table 9:** Example of contingency table for the CS taxonomy's dimension "Geographic scope" with dichotomized cluster allocation vector in order to test the (in)dependence of the first cluster (Innovation oriented port eco-systems) from the remaining CS for the "Geographic scope” dimension (see for comparison Appendix Table 8); totals in bold

		Geographic scope		
		Local	Regional	National
Cluster	1 - Innovation oriented port eco-systems	**0**	**2**	**6**
	2 - B2B focused CS & 3 - Non-profit CS & 4 - Non-specialized Single Windows	3 + 0 + 2 = **5**	3 + 8 + 13 = **24**	0 + 5 + 2 = **7**

**Table 10:** Contingency table analysis results for taxonomy dimensions

Contingency table dimensions	Chi-Sq^+^	p-Value (Chi-Sq)^+^	Cramer's V^+^	Corrected Cramer's _V_ +	p-Value (Fisher exact test)^+^
Market ↔ Geographic Scope	30,9	0,014 *	0,59	0,34	0,035 *
Market ↔ Modes of transport	146,2	0 ***	0,69	0,54	0,03 *
Market ↔ Communication services	75,0	0 ***	0,75	0,55	0,001 ***
Market ↔ Value-added services	76,5	0,036 *	0,50	0,25	0,327
Market ↔ Extended services	60,8	0,101	0,48	0,21	0,041 *
Market ↔ Platform origin	14,7	0,066 *	0,58	0,27	0,07 *
Market ↔ Interface	31,2	0,971	0,34	0,00	0,690
Market ↔ Data analytics	36,3	0,274	0,45	0,13	0,395
Market ↔ Data security	12,9	0,117	0,54	0,23	0,155
Market ↔ Data governance	21,5	0,006 **	0,70	0,39	0,190
Market ↔ Role of governmental actors	40,9	0,017 *	0,56	0,31	0,045 *
Market ↔ Decisional openness	13,3	0,103	0,55	0,24	0,130
Market ↔ Complementor openness	71,0	0 ***	0,73	0,53	0,033 *
Market ↔ Business objective of operator	13,4	0,098 *	0,55	0,25	0,061 *
Market ↔ Funding	17,8	0,364	0,45	0,11	0,358
Market ↔ Funding continuity	12,3	0,702	0,37	0,00	0,607
Market ↔ Payment model	16,7	0,818	0,36	0,00	0,843
Geographic Scope ↔ Modes of transport	18,7	0,283	0,46	0,19	0,336
Geographic Scope ↔ Communication services	15,4	0,052 *	0,42	0,27	0,108
Geographic Scope ↔ Value-added services	14,2	0,434	0,40	0,00	0,332
Geographic Scope ↔ Extended services	11,3	0,507	0,36	0,00	0,657
Geographic Scope ↔ Platform origin	9,2	0,01 *	0,46	0,29	0,016 *
Geographic Scope ↔ Interface	8,9	0,710	0,32	0,00	0,728
Geographic Scope ↔ Data analytics	15,2	0,056 *	0,42	0,23	0,171
Geographic Scope ↔ Data security	4,8	0,09 *	0,33	0,18	0,124
Geographic Scope ↔ Data governance	2,4	0,295	0,24	0,07	0,412
Geographic Scope ↔ Role of governmental actors	8,0	0,239	0,30	0,12	0,349
Geographic Scope ↔ Decisional openness	5,5	0,063 *	0,35	0,20	0,104
Geographic Scope ↔ Complementor openness	11,3	0,078 *	0,36	0,20	0,056 *
Geographic Scope ↔ Business objective of operator	6,2	0,045 *	0,38	0,22	0,054 *
Geographic Scope ↔ Funding	2,8	0,631	0,18	0,00	0,618
Geographic Scope ↔ Funding continuity	11,8	0,055 *	0,37	0,25	0,073 *
Geographic Scope ↔ Payment model	3,0	0,790	0,19	0,00	0,785
Modes of transport ↔ Communication services	30,1	0,562	0,48	0,23	0,615
Modes of transport ↔ Value-added services	82,0	0,013 *	0,52	0,33	0,099 *
Modes of transport ↔ Extended services	49,8	0,403	0,43	0,16	0,323
Modes of transport ↔ Platform origin	10,8	0,216	0,49	0,20	0,199
Modes of transport ↔ Interface	39,1	0,815	0,39	0,00	0,400
Modes of transport ↔ Data analytics	27,8	0,678	0,40	0,00	0,394
Modes of transport ↔ Data security	11,1	0,198	0,50	0,21	0,220
Modes of transport ↔ Data governance	8,0	0,435	0,43	0,10	0,242
Modes of transport ↔ Role of governmental actors	32,5	0,116	0,50	0,26	0,152
Modes of transport ↔ Decisional openness	16,3	0,038 *	0,61	0,32	0,014 *
Modes of transport ↔ Complementor openness	57,6	0 ***	0,66	0,46	0,083 *
Modes of transport ↔ Business objective of operator	6,1	0,632	0,37	0,00	0,810
Modes of transport ↔ Funding	16,0	0,472	0,43	0,11	0,581
Modes of transport ↔ Funding continuity	23,9	0,134	0,52	0,27	0,016 *
Modes of transport ↔ Payment model	30,4	0,235	0,48	0,23	0,254
Communication services ↔ Value-added services	30,1	0,357	0,48	0,23	0,065 *
Communication services ↔ Extended services	23,9	0,470	0,43	0,18	0,194
Communication services ↔ Platform origin	15,3	0,004 **	0,59	0,37	0,002 **
Communication services ↔ Interface	21,5	0,607	0,40	0,14	0,216
Communication services ↔ Data analytics	25,5	0,061 *	0,44	0,28	0,031 *
Communication services ↔ Data security	8,6	0,073 *	0,44	0,25	0,086 *
Communication services ↔ Data governance	2,2	0,700	0,22	0,00	0,450
Communication services ↔ Role of governmental actors	30,7	0,002 **	0,48	0,36	0,012 *
Communication services ↔ Decisional openness	3,3	0,516	0,27	0,05	0,773
Communication services ↔ Complementor openness	10,9	0,538	0,29	0,10	0,337
Communication services ↔ Business objective of operator	4,4	0,354	0,32	0,12	0,478
Communication services ↔ Funding	6,7	0,578	0,28	0,07	0,608
Communication services ↔ Funding continuity	5,7	0,675	0,25	0,00	0,662
Communication services ↔ Payment model	10,5	0,596	0,28	0,09	0,594
Value-added services ↔ Extended services	23,2	0,992	0,30	0,00	0,951
Value-added services ↔ Platform origin	5,2	0,633	0,34	0,00	0,724
Value-added services ↔ Interface	49,8	0,191	0,43	0,16	0,285
Value-added services ↔ Data analytics	27,6	0,485	0,40	0,00	0,138
Value-added services ↔ Data security	11,0	0,137	0,50	0,21	0,139
Value-added services ↔ Data governance	1,6	0,978	0,19	0,00	1,000
Value-added services ↔ Role of governmental actors	20,5	0,489	0,39	0,00	0,410
Value-added services ↔ Decisional openness	8,4	0,297	0,44	0,12	0,230
Value-added services ↔ Complementor openness	11,1	0,961	0,29	0,00	0,829
Value-added services ↔ Business objective of operator	8,1	0,326	0,43	0,10	0,318
Value-added services ↔ Funding	17,5	0,265	0,45	0,16	0,315
Value-added services ↔ Funding continuity	11,8	0,670	0,37	0,00	0,553
Value-added services ↔ Payment model	17,5	0,663	0,36	0,00	0,686
Extended services ↔ Platform origin	6,0	0,418	0,37	0,00	0,500
Extended services ↔ Interface	32,9	0,615	0,35	0,00	0,588
Extended services ↔ Data analytics	31,6	0,136	0,42	0,19	0,312
Extended services ↔ Data security	12,4	0,053 *	0,53	0,27	0,085 *
Extended services ↔ Data governance	3,5	0,747	0,28	0,00	0,549
Extended services ↔ Role of governmental actors	21,3	0,263	0,40	0,13	0,240
Extended services ↔ Decisional openness	13,4	0,037 *	0,55	0,29	0,027 *
Extended services ↔ Complementor openness	25,1	0,123	0,44	0,20	0,225
Extended services ↔ Business objective of operator	5,9	0,435	0,37	0,00	0,453
Extended services ↔ Funding	9,2	0,679	0,32	0,00	0,705
Extended services ↔ Funding continuity	6,7	0,862	0,28	0,00	0,840
Extended services ↔ Payment model	13,9	0,718	0,32	0,00	0,764
Platform origin ↔ Interface	4,6	0,592	0,32	0,00	0,648
Platform origin ↔ Data analytics	1,5	0,827	0,18	0,00	0,730
Platform origin ↔ Data security	1,0	0,310	0,15	0,01	0,311
Platform origin ↔ Data governance	0,0	1,000	0,00	0,00	1,000
Platform origin ↔ Role of governmental actors	7,6	0,055 *	0,42	0,23	0,033 *
Platform origin ↔ Decisional openness	5,0	0,025 *	0,34	0,21	0,009 **
Platform origin ↔ Complementor openness	1,4	0,705	0,18	0,00	0,885
Platform origin ↔ Business objective of operator	0,3	0,592	0,08	0,00	0,486
Platform origin ↔ Funding	0,6	0,756	0,12	0,00	0,817
Platform origin ↔ Funding continuity	4,2	0,202	0,31	0,16	0,231
Platform origin ↔ Payment model	2,1	0,612	0,22	0,00	0,649
Interface ↔ Data analytics	44,0	0,008 **	0,50	0,31	0,086 *
Interface ↔ Data security	5,4	0,496	0,35	0,00	0,568
Interface ↔ Data governance	1,8	0,938	0,20	0,00	0,584
Interface ↔ Role of governmental actors	14,4	0,703	0,33	0,00	0,566
Interface ↔ Decisional openness	6,1	0,409	0,37	0,00	0,497
Interface ↔ Complementor openness	8,2	0,976	0,25	0,00	0,854
Interface ↔ Business objective of operator	9,8	0,133	0,47	0,21	0,125
Interface ↔ Funding	8,8	0,710	0,32	0,00	0,817
Interface ↔ Funding continuity	6,7	0,840	0,28	0,00	0,763
Interface ↔ Payment model	18,7	0,476	0,38	0,04	0,457
Data analytics ↔ Data security	8,1	0,087 *	0,43	0,22	0,162
Data analytics ↔ Data governance	6,5	0,166	0,38	0,17	0,239
Data analytics ↔ Role of governmental actors	10,4	0,577	0,28	0,00	0,500
Data analytics ↔ Decisional openness	1,0	0,903	0,15	0,00	1,000
Data analytics ↔ Complementor openness	24,9	0,015 *	0,43	0,28	0,028 *
Data analytics ↔ Business objective of operator	6,2	0,184	0,38	0,16	0,203
Data analytics ↔ Funding	8,1	0,491	0,30	0,00	0,475
Data analytics ↔ Funding continuity	14,4	0,149	0,40	0,22	0,326
Data analytics ↔ Payment model	10,7	0,577	0,29	0,00	0,520
Data security ↔ Data governance	1,1	0,284	0,16	0,00	0,136
Data security ↔ Role of governmental actors	2,8	0,430	0,25	0,00	0,481
Data security ↔ Decisional openness	2,3	0,133	0,23	0,00	0,071 *
Data security ↔ Complementor openness	9,5	0,024 *	0,46	0,27	0,052 *
Data security ↔ Business objective of operator	0,0	1,000	0,00	0,00	1,000
Data security ↔ Funding	2,5	0,368	0,24	0,00	0,396
Data security ↔ Funding continuity	0,8	0,690	0,14	0,00	0,764
Data security ↔ Payment model	1,3	0,750	0,17	0,00	0,798
Data governance ↔ Role of governmental actors	1,2	0,746	0,17	0,00	1,000
Data governance ↔ Decisional openness	0,2	0,693	0,06	0,00	0,318
Data governance ↔ Complementor openness	21,5	0 ***	0,70	0,39	0,059 *
Data governance ↔ Business objective of operator	0,0	1,000	0,00	0,00	1,000
Data governance ↔ Funding	2,3	0,327	0,23	0,00	0,484
Data governance ↔ Funding continuity	0,6	0,750	0,11	0,00	1,000
Data governance ↔ Payment model	0,7	0,884	0,12	0,00	1,000
Role of governmental actors ↔ Decisional openness	1,2	0,749	0,17	0,00	0,820
Role of governmental actors ↔ Complementor openness	18,0	0,035 *	0,45	0,30	0,011 *
Role of governmental actors ↔ Business objective of operator	24,7	0 ***	0,75	0,50	0 ***
Role of governmental actors ↔ Funding	15,9	0,033 *	0,43	0,28	0,021 *
Role of governmental actors ↔ Funding continuity	4,8	0,581	0,23	0,00	0,575
Role of governmental actors ↔ Payment model	15,6	0,133	0,42	0,27	0,122
Decisional openness ↔ Complementor openness	0,9	0,818	0,15	0,00	0,849
Decisional openness ↔ Business objective of operator	0,0	1,000	0,00	0,00	1,000
Decisional openness ↔ Funding	0,8	0,696	0,14	0,00	0,762
Decisional openness ↔ Funding continuity	1,3	0,571	0,17	0,00	0,764
Decisional openness ↔ Payment model	1,0	0,802	0,15	0,00	0,845
Complementor openness ↔ Business objective of operator	3,7	0,298	0,29	0,08	0,225
Complementor openness ↔ Funding	6,8	0,380	0,28	0,07	0,330
Complementor openness ↔ Funding continuity	5,1	0,544	0,24	0,00	0,467
Complementor openness ↔ Payment model	4,8	0,830	0,19	0,00	0,761
Business objective of operator ↔ Funding	13,0	0,008 **	0,54	0,26	0,008 **
Business objective of operator ↔ Funding continuity	1,4	0,523	0,18	0,00	0,661
Business objective of operator ↔ Payment model	8,4	0,079 *	0,44	0,12	0,082 *
Funding ↔ Funding continuity	8,2	0,156	0,31	0,00	0,178
Funding ↔ Payment model	45,2	0 ***	0,72	0,51	0 ***
Funding continuity ↔ Payment model	11,1	0,135	0,50	0,30	0,128

^+^ - Based on average for IMP data

## References

[CR1] 1-Stop. (2018). *1-Stop TABS - Manila, two years on*. https://www.youtube.com/watch?v=-IpggSVPco8. Accessed 17 Mar 2021.

[CR2] 1-Stop. (2019). *1-Stop | API Programme. *https://www.1-stop.biz/api/. Accessed 17 Mar 2021.

[CR3] 1-Stop. (2020a). *1-Stop—Solutions | Port Products and Solutions | API Programme | Vehicle booking system.* https://www.1-stop.biz/solutions/ -- https://www.1-stop.biz/api/ -- https://www.1-stop.biz/wp-content/uploads/2018/04/1-Stop-VBS-Brochure_A4-Portrait_FA-Web.pdf. Accessed 17 Mar 2021.

[CR4] 1-Stop. (2020b). *DirectPay: A new optional payment solution by 1-Stop. *https://www.1-stop.biz/directpay-a-new-optional-payment-solution-by-1-stop/. Accessed 17 Mar 2021.

[CR5] 1-Stop TABS. (2015). *About TABS*. TABS. http://1-stop.com.ph/about/. Accessed 17 Mar 2021.

[CR6] Abendroth, J., Riefle, L., & Benz, C. (2021). Opening the Black Box of Digital B2B Co-Creation Platforms: A Taxonomy. In: Ahlemann F., Schütte R., Stieglitz S. (eds) Innovation Through Information Systems. *WI 2021. Lecture Notes in Information Systems and Organisation*, vol 47. Springer, Cham. 10.1007/978-3-030-86797-3_39

[CR7] Abramson MA, Morin TL (2003). E-government 2003.

[CR8] Abu Dhabi Ports. (2018). *Port Circular 03/2018*. https://www.adports.ae/wp-content/uploads/2018/07/CIRC03.2018.pdf. Accessed 15 Mar 2021.

[CR9] Acquah-Bentil, E. (2015). *Statistical Analysis of the Impact of the Gcnet on Revenue Performance in Ghana:(A Case Study of Tema Port)* [PhD Thesis]. University of Ghana.

[CR10] Adaba GB, Rusu L (2014). E-trade Facilitation in Ghana: A capability approach perspective. The Electronic Journal of Information Systems in Developing Countries.

[CR11] Advent Intermodal Solutions. (2020). *EModal Community Portal—General—Learning Materials—Port Solutions*. https://www.adventintermodal.com/home/solutions/emodal -- https://learning.emodal.com/ecp2/#/menu/5f84c376d1d2ec22e2fe5db4 -- https://www.adventintermodal.com/solutions/port-solutions. Accessed 15 Mar 2021.

[CR12] Agarwal N, Brem A (2015). Strategic business transformation through technology convergence: Implications from General Electric’s industrial internet initiative. International Journal of Technology Management.

[CR13] Agyemang, S. K. (2016). *Gcnet and the Facilitation of International Trade at the Port of Tema* [PhD Thesis]. University of Ghana.

[CR14] Akande O, Li F, Reiter J (2017). An empirical comparison of multiple imputation methods for categorical data. The American Statistician.

[CR15] Amditis, A. (2020). *Building an innovation culture in large State Owned Enterprises*. HCAP - InnovationNetwork. https://hcap.labonline.gr/event-3/. Accessed 15 Mar 2021.

[CR16] Ancharaz, S. D. (2018). Best practices in digital customs in east and southern africa – a critical assessment of the success story of mra. 52. *WCO ESA ROCB Conference 2017.*

[CR17] Antwerp Port Authority. (2015). *Product Sheet eServices_LR.pdf*. https://www.portofantwerp.com/sites/default/files/Product%20Sheet%20eServices_LR.pdf. Accessed 15 Mar 2021.

[CR18] Apple App Store. (2020). *e-puertobilbao*. Apple App Store. https://apps.apple.com/us/app/preavisos-e-puertobilbao/id1247449201. Accessed 01 Apr 2021.

[CR19] Arduino G, Aronietis R, Crozet Y, Frouws K, Ferrari C, Guihery L, Kapros S, Kourounioti L, Laroche F, Lambrou M, Lloyd M, Polydoropoulou A, Roumboutsos A, Van de Voorde E, Vanelslander T (2013). How to turn an innovative concept into a success? An application to seaport- related innovation. Research in Transportation Economics.

[CR20] Arkima, A. (2017). *Portnet– National Single Windowimplementationin Finland* [UNECE]. https://www.unece.org/fileadmin/DAM/cefact/cf_forums/2017_Geneva/PPTs/LOCODE/S2.2-Experiences_Finland.pdf. Accessed 15 Mar 2021.

[CR21] Arkima, A. (2019). *Finland* (Single Window Repository). UNECE. https://www.unece.org/fileadmin/DAM/cefact/single_window/sw_cases/Download/Finland.pdf. Accessed 15 Mar 2021.

[CR22] Autorità Portuale di Ravenna. (2020). *Port Community System del Porto di Ravenna*. http://www.port.ravenna.it/wp-content/uploads/storico/Configurazione%20PCS.pdf. Accessed 17 Mar 2021.

[CR23] Barnett, C. (2018). *Brussels Airport: Brussels’ strategy offers path for congested air cargo hubs*. JoC Online. https://www.joc.com/air-cargo/international-air-freight/brussels%E2%80%99-strategy-offers-path-congested-air-cargo-hubs_20181119.html. Accessed 18 Mar 2021.

[CR24] Baron M-L, Mathieu H (2013). PCS interoperability in Europe: A market for PCS operators?. International Journal of Logistics Management.

[CR25] Basagaña X, Barrera-Gómez J, Benet M, Antó JM, Garcia-Aymerich J (2013). A Framework for Multiple Imputation in Cluster Analysis. American Journal of Epidemiology.

[CR26] Batyrshin IZ, Kubysheva N, Solovyev V, Villa-Vargas LA (2016). Visualization of Similarity Measures for Binary Data and 2x2 Tables. Computación y Sistemas.

[CR27] Bellos, I. (2020).* Not everyone is happy with digital system in Piraeus*. EKathimerini.Com. https://www.ekathimerini.com/economy/249903/not-everyone-is-happy-with-digital-system-in-piraeus/. Accessed 15 Mar 2021.

[CR28] Ben-Moshe, G. (2017). *Israel Port Community System*. http://www.israports.org.il/en/IPCS/Documents/%D7%9E%D7%A6%D7%92%D7%AA%20%D7%AA%D7%A1%D7%A7%20%D7%99%D7%9D.pdf. Accessed 19 Mar 2021.

[CR29] Bergsma W (2013). A bias-correction for Cramér’s V and Tschuprow’s T. Journal of the Korean StatisticalSociety.

[CR30] Bilbaoport. (2020). *The Port Authority*. https://www.bilbaoport.eus/en/the-port-authority/. Accessed 18 Mar 2021.

[CR31] Bivona E, Cosenz F (2021). Designing a Multi-Sided Platform business model assessment framework: A Dynamic Performance Management perspective. Systems Research and Behavioral Science.

[CR32] Blaschke, M., Haki, K., Aier, S., & Winter, R. (2019). Taxonomy of digital platforms: A platform architecture perspective. *Wirtschaftsinformatik 2019 Proceedings*. https://aisel.aisnet.org/wi2019/track06/papers/3. Accessed 22 May 2021.

[CR33] Blasquez, P. (2020).* Planning for Vaccine Distribution: DFW Airport, American Airlines Make Dallas-Fort Worth an Ideal Gateway . *Dallas Innovates. https://dallasinnovates.com/planning-for-vaccine-distribution-dfw-airport-american-airlines-make-dallas-fort-worth-an-ideal-gateway/. Accessed 15 Mar 2021.

[CR34] Bock, M., & Wiener, M. (2017). Towards a Taxonomy of Digital Business Models - Conceptual Dimensions and Empirical Illustrations. *ICIS 2017 Proceedings*. https://aisel.aisnet.org/icis2017/Strategy/Presentations/19. Accessed 03 Dec 2020.

[CR35] Boriah, S., Chandola, V., & Kumar, V. (2008). Similarity Measures for Categorical Data: A Comparative Evaluation. *Proceedings of the 2008 SIAM International Conference on Data Mining*, 243–254. 10.1137/1.9781611972788.22

[CR36] Brett, D. (2020). *Swissport expands cargo capacity in Frankfurt.* Air Cargo News. https://www.aircargonews.net/region/europe/swissport-expands-cargo-capacity-in-frankfurt/. Accessed 11 Mar 2021.

[CR37] Brussels Airport. (2021). *BRUCloud*. https://brucloud.com/about/brucloud. Accessed 13 Mar 2021.

[CR38] Bunker Ports News Worldwide. (2019). *SOGET and the start-up Click2Rail sign a global partnership agreement*. http://www.bunkerportsnews.com//News.aspx?ElementID=db08b8f5-0d2a-4dec-807c-b40c0a9ceccd. Accessed 16 Mar 2021.

[CR39] CAAS. (2018). *Slow start for Heathrow CargoCloud.* CAAS - Cargo Airports & Airline Services. https://www.caasint.com/issue-article/slow-start-for-heathrow-cargocloud/. Accessed 14 Mar 2021.

[CR40] Cargonaut. (2021). *Cargonaut—Services*. Cargonaut Services. https://cargonaut.nl/services/. Accessed 15 Mar 2021.

[CR41] Carlan V, Sys C, Calatayud A, Vanelslander T (2018). Digital innovation in maritime supply chains: Experiences from Northwestern Europe.

[CR42] Carlan V, Sys C, Vanelslander T (2016). How port community systems can contribute to port competitiveness: Developing a cost–benefit framework. Research in Transportation Business & Management.

[CR43] Carlin, J. B., Li, N., Greenwood, P., & Coffey, C. (2003). Tools for analyzing multiple imputed datasets. *The Stata Journal,**3*(3), 226–244. 10.1177/1536867X0300300302

[CR44] CCS-UK. (2021). *CCS-UK system*. The CCS-UK Cargo Community User Group Ltd. https://www.ccs-uk-ug.org/index.php/products/ccs-uk-system. Accessed 12 Mar 2021.

[CR45] CEI.BA. (2021). *AP+ Guadeloupe—First Cargo Community System in the Caribbean.* http://www.ceiba-gp.com/products/?lang=en. Accessed 20 Mar 2021.

[CR46] Chandra DR, van Hillegersberg J (2018). Governance of inter-organizational systems: A longitudinal case study of Rotterdam’s Port Community System. Ijispm-International Journal of Information Systems and Project Management.

[CR47] Chandra, D. R., & van Hillegersberg, J. (2019). Creating competitive advantage for air freight communities using a cargo community system: A case study in Amsterdam Schiphol airport. *25th Americas Conference on Information Systems, AMCIS 2019*.

[CR48] Chen D, Zhao H (2012). Data security and privacy protection issues in cloud computing. 2012 International Conference on Computer Science and Electronics Engineering.

[CR49] Cheng, M. C., & Wang, J. J. (2016). An integrative approach in measuring hub-port supply chain performance: Potential contributions of a logistics and transport data exchange platform. *Case Studies on Transport Policy,**4*(2), 150–160. 10.1016/j.cstp.2016.03.001

[CR50] Choudhury, A., & Kosorok, M. R. (2020). Missing Data Imputation for Classification Problems. *ArXiv:2002.10709 [Cs, Stat]*. http://arxiv.org/abs/2002.10709. Accessed 20 May 2021.

[CR51] Christiaanse E, Damsgaard J, Khosrow-Pour M (2006). Success and failure in building electronic infrastructures in the Air Cargo Industry: A comparison of the Netherlands and Hong Kong SAR. Cases on information technology and organizational politics & culture.

[CR52] CNS. (2021). *Community Network Services Ltd | CNS. *https://www.cnsonline.co.uk/home/. Accessed 15 Mar 2021.

[CR53] Constante, J. M. (2019). *International Case Studies and Good Practices for Implementing Port Community Systems. Publications*. Inter-American Development Bank. https://publications.iadb.org/publications/english/document/International_case_studies_and_good_practices_for_implementing_Port_Community_Systems_en_en.pdf. Accessed 15 Mar 2021.

[CR54] Courea, E. (2020). *Post-Brexit border checks to cost businesses £13 billion*. https://www.thetimes.co.uk/article/post-brexit-border-checks-to-cost-businesses-13-billion-3r5w8hhxk. Accessed 12 Mar 2021.

[CR55] Cramér, H. (1946). The two-dimensional case. In *Mathematical Methods of Statistics* (p. 575). Princeton University Press.

[CR56] Crimsonlogic. (2018). *Sharing our experience in implementing Port Community System*. https://www.apn.gob.pe/site/files/URRI34534534583945898934857345/05-PCS-Lima.pdf. Accessed 15 Mar 2021.

[CR57] Cusumano, M. A., Gawer, A., & Yoffie, D. B. (2019). *The Business of Platforms: Strategy in the Age of Digital Competition, Innovation, and Power* (Illustrated Edition). Harper Business.

[CR58] Cyberark. (2020). *THE CYBERARK DIGITAL VAULT: BUILT FOR SECURITY*. https://www.cyberark.com/resources/product-datasheets/the-cyberark-digital-vault-built-for-security. Accessed 11 Mar 2021.

[CR59] DAKOSY. (2020a). *About us—DAKOSY Datenkommunikationssystem AG*. https://www.dakosy.de/en/about-us. Accessed 16 Mar 2021.

[CR60] DAKOSY. (2020b). *FAIR@Link*. https://www.dakosy.de/loesungen/cargo-communications/air-cargo-community-system/fairlink/. Accessed 16 Mar 2021.

[CR61] DAKOSY. (2020c). *Port Community System—DAKOSY Datenkommunikationssystem AG*. https://www.dakosy.de/loesungen/cargo-communications/port-community-system. Accessed 17 Mar 2021.

[CR62] Damsgaard J (1999). Global Logistics System Asia Co., Ltd. Journal of Information Technology.

[CR63] Davison, R. M., Wagner, C., & Ma, L. C. (2005). From government to e-government: A transition model. *Information Technology & People, 18*(3), 280–299. 10.1108/09593840510615888

[CR64] dbh Logistics IT AG. (2011). *Datenschnittstelle zur Bremer Hafentelematik (BHT)*. yumpu. https://www.yumpu.com/de/document/read/46407759/bremer-hafentelemat-bremer-hafentelematik-bht-kis-dbh. Accessed 15 Mar 2021.

[CR65] dbh Logistics IT AG. (2020).* Port Management.* https://www.dbh.de/en/port-management/. Accessed 15 Mar 2021.

[CR66] de Blic, Y., Hoene, A., Karaarslan, S., Kreukniet, N., Singh, P., Szymiczek, M., Tsiochantari, G., & Wernert, J. (2018). IT Technologies for Inland Waterway Transport (Report NWE574 Smart Tracking Data Network for Shipment by Inland Waterway: Smart Track 4 Waterway. Deliverable No. 1.1 State-of-the-Art).Duisburg-Essen Publication Online. 10.17185/duepublico/47501

[CR67] De la Guia, J. M. G. (2013). *The Port of Montreal’s advantages. *Port of Montreal. https://portofmontreal.ca/en/a-system-for-all-december2012.html. Accessed 17 Mar 2021.

[CR68] de Langen PW, Heij C (2014). Corporatisation and performance: A literature review and an analysis of the performance effects of the corporatisation of port of Rotterdam authority. Transport Reviews.

[CR69] de Langen PW (2005). Trends and opportunities for the long-term development of Rotterdam’s Port complex. Coastal Management.

[CR70] de Reuver M, Sørensen C, Basole RC (2018). The digital platform: A research agenda. Journal of Information Technology.

[CR71] de Sena Pedro Salvador, A. (2017). *Tese de Mestrado de Antónia Salvador-Importância das características dos Sistemas Comunitários Portuários (PCS - Port Community Systems) no desempenho dos portos Versão Final.pdf*. Escola Superior de Ciencias Empresariais.

[CR72] Delucchi KL (1983). The use and misuse of chi-square: Lewis and Burke revisited. Psychological Bulletin.

[CR73] Desormeaux, H. (2016). *Abu Dhabi Ports implements digital vessel management system.* FreightWaves. https://www.freightwaves.com/news/abu-dhabi-ports-implements-digital-vessel-management-system. Accessed 17 Mar 2021.

[CR74] Devex. (2021). *Ghana Community Network Services Limited (GCNeT)*. https://www.devex.com/organizations/ghana-community-network-services-limited-gcnet-39674. Accessed 11 Mar 2021.

[CR75] Di Vaio A, Varriale L (2020). Digitalization in the sea-land supply chain: Experiences from Italy in rethinking the port operations within inter-organizational relationships. Production Planning & Control.

[CR76] Diaz, M. (2003). Port Community System–A Key Component of the Future Vision for Cargo and Port Securi-7. Ty. *Government Supply Chain Blue Papers, Valencia*.

[CR77] DP World. (2020). *Dubai Trade*. http://www.dubaitrade.ae/. Accessed 09 Mar 2021.

[CR78] DP World Southampton. (2020). *Community Network Services Ltd. *CNS. https://www.cnsonline.co.uk/home/#/content/about/approvedsoftwarehouses. Accessed 15 Mar 2021.

[CR79] DPCS. (2020). *DPCS – Djibouti Port Community Systems*. https://www.dpcs.dj/TFBPCS/login/explore-trade.jsp. Accessed 20 Mar 2021.

[CR80] DTledger. (2020). *Blockchain Platform dltledgers Joins Singapore Government’s Networked Trade Platform.* Dltledgers. https://dlt.sg/blockchain-platform-dltledgers-joins-singapore-governments-networked-trade-platform/. Accessed 21 Mar 2021.

[CR81] DTTN. (2021). *DTTN Services*. http://www.hk-dttn.com/services/english/optimizeyourtradelogisticandfinanceprocesseswiththedttn.html. Accessed 15 Mar 2021.

[CR82] Dubai Trade. (2016). *About Dubai Trade*. http://dtcms.cachefly.net/jdownloads/download-center/about/about+dubai+trade.pdf. Accessed 09 Mar 2021.

[CR83] Durach CF, Kembro J, Wieland A (2017). A new paradigm for systematic literature reviews in supply chain management. Journal of Supply Chain Management.

[CR84] DVZ. (2017). *Hafen Antwerpen erweitert IT-Plattform für Rhein Ports*. https://www.dvz.de/rubriken/land/binnenschifffahrt/detail/news/hafen-antwerpen-erweitert-it-plattform-fuer-rhein-ports.html. Accessed 15 Mar 2021.

[CR85] DVZ. (2020). *LKW-Transporte nach Großbritannien sollen auch nach einem No Deal im Brexit reibungslos laufen können.* Deutsche Verkehrs-Zeitung. https://www.dvz.de/rubriken/management-recht/zoll/detail/news/brexit-reibungsloser-transport-auch-bei-einem-no-deal.html. Accessed 28 Mar 2021.

[CR86] Ebrahim Z, Irani Z (2005). E-government adoption: Architecture and barriers. Business Process Management Journal.

[CR87] Efron, B. (1994). Missing data, imputation, and the bootstrap. *Journal of the American Statistical Association,**89*(426), 463–475.

[CR88] Eisenhardt, K. M. (1989). Building theories from case study research. *Academy of Management Review,**14*(4), 532–550. 10.5465/amr.1989.4308385

[CR89] El Sawy, O. A., & Pereira, F. (2013). *Business modelling in the dynamic digital space: An ecosystem approach*. Springer. Berlin, Heidelberg. 10.1007/978-3-642-31765-1

[CR90] Elbert R, Tessmann R (2021). Port Community Systems-Supply Chain App stores of the future?. Internationales Verkehrswesen.

[CR91] EPCSA. (2011). *How To Develop A Port Community System*. https://www.unece.org/fileadmin/DAM/trade/Trade_Facilitation_Forum/BkgrdDocs/HowToDevelopPortCommunitySystem-EPCSAGuide.pdf. Accessed 01 Jan 2021.

[CR92] e-puertobilbao. (2016). *EDI guide e-puerto bilbao*. https://www.epb.biz/wp-content/uploads/2018/05/EPB_Coprar_v1.2.3_D95B_CARGA_FULL.pdf. Accessed 15 Mar 2021.

[CR93] e-puertobilbao. (2020). *What is e-puertobilbao*. https://www.epuertobilbao.com/en/what-is-e-puertobilbao/. Accessed 15 Mar 2021.

[CR94] Essay UK. (2020). *An Evaluation Of The Impact Of A Port Community System On The Logistics Supply Chain Of Mauritius—Free Business Essay—Essay UK*. https://www.essay.uk.com/free-essays/business/evaluation-impact-port-community-system.php. Accessed 21 Mar 2021.

[CR95] Evans, D. S. (2008). *Competition and Regulatory Policy for Multi-Sided Platforms with Applications to the Web Economy* (SSRN Scholarly Paper ID 1090368). Social Science Research Network. 10.2139/ssrn.1090368

[CR96] Evans DS, Schmalensee R (2016). Matchmakers: The new economics of multisided platforms.

[CR97] Fanti, M. P., Ukovich, W., Berardi, A., Di Pierro, B., & Giansante, C. (2020). *Existing platforms and technical requirements for the architecture of the federated ecosystem* (D2.2.1; FENIX Network, p. 96). https://fenix-network.eu/wp-content/uploads/2020/07/FENIX-Deliverable-D2.2.1_v2.0_FINAL.pdf. Accessed 01 Apr 2021.

[CR98] FAQ Logistique. (2018). *MGI and SOGET present the convergence work of Ci5 and S) ONE*. Portail Logistique, Transport et Supply Chain https://www.faq-logistique.com/CP20180322-MGI-SOGET-Convergence-Nationale-CCS.htm. Accessed 17 Mar 2021.

[CR99] Farahani, R. Z., Rezapour, S., Drezner, T., & Fallah, S. (2014). Competitive supply chain network design: An overview of classifications, models, solution techniques and applications. *Omega,**45*, 92–118. 10.1016/j.omega.2013.08.006

[CR100] Fedi L, Lavissiere A, Russell D, Swanson D (2019). The facilitating role of IT systems for legal compliance: The case of port community systems and container Verified Gross Mass (VGM). Supply Chain Forum: International Journal.

[CR101] Feld H (2019). The case for the digital platform act: Market structure and regulation of digital platforms.

[CR102] Finance T (2012). Dubai Trade reaches milestone. Trade Finance.

[CR103] Finch H (2005). Comparison of distance measures in cluster analysis with dichotomous data. Journal of Data Science.

[CR104] Foss NJ, Saebi T (2017). Fifteen years of research on business model innovation: How far have we come, and where should we go?. Journal of Management.

[CR105] Foucault, C. (2019). *Issue 36: January – April 2019—Caribbean Maritime—Journal of the Caribbean Shipping Association* [Interview]. https://www.caribbean-maritime.com/index.php/latest22/issue-36-january-april-2019.html?start=1. Accessed 18 Mar 2021.

[CR106] Furio S, Bergqvist R, Wilmsmeier G, Cullinane K (2013). Port community systems in maritime and rail transport integration: The case of Valencia, Spain. Dry ports – a global perspective challenges and developments in serving Hinterlands.

[CR107] Gawer A (2014). Bridging differing perspectives on technological platforms: Toward an integrative framework. Research Policy.

[CR108] Giannopoulos, G. A. (2008). *ICT systems and services for port operation and management:* (p. 22). UNECE. https://unece.org/DAM/trans/doc/2008/wp5/GE1_Piraeus_Item3_Giannopoulos.pdf. Accessed 01 Mar 2021.

[CR109] Giannopoulos GA (2009). Towards a European ITS for freight transport and logistics: Results of current EU funded research and prospects for the future. European Transport Research Review.

[CR110] Gil-Campos, T. L. (2016). *Impacts of Information Technology (IT) Systems on the Efficiency of Empty-Container Park Operations for the Port of Melbourne* [Master Thesis] http://vuir.vu.edu.au/31013/3/GIL-CAMPOS%20Teresa-thesis_nosignature.pdf. Accessed 01 Feb 2021.

[CR111] Gimpel H, Rau D, Röglinger M (2018). Understanding FinTech start-ups – a taxonomy of consumer- oriented service offerings. Electronic Markets.

[CR112] Gladiator, D. (2020). *Erfahrungsbericht vom Frankfurter Flughafen: Wo LKW vom Himmel fallen. *Deutsche Verkehrs-Zeitung. https://www.dvz.de/rubriken/luft/detail/news/wo-lkw-vom-himmel-fallen.html. Accessed 15 Mar 2021.

[CR113] Google Play. (2021). *PLISM3.0. *https://play.google.com/store/apps/details?id=com.klnet.plism3.plsim3&hl=ar&gl=US. Accessed 03 Apr 2021.

[CR114] Gordon JRM, Lee P-M, Lucas HC (2005). A resource-based view of competitive advantage at the Port of Singapore. The Journal of Strategic Information Systems.

[CR115] Graham JW, Olchowski AE, Gilreath TD (2007). How many imputations are really needed? Some practical clarifications of multiple imputation theory. Prevention Science: The Official Journal of the Society for Prevention Research.

[CR116] Grandjean, J. (2019). *Discreet and essential EDI* [Interview]. https://www.mgi-ci5.com/en/mgi-talent-discreet-and-essential-edi/. Accessed 18 Mar 2021.

[CR117] Gregor, S. (2006). The nature of theory in information systems. *MIS Quarterly, 30*(3), 611–642. 10.2307/25148742

[CR118] Grinias, K., Iakovou, E., Vlachos, D., Gkikas, A., Tsoukos, G., & Bizakis, A. (2015). The Strategic Role of Port Community Systems for enhancing Business Operations and Productivity: The Case of the Port Authority of Igoumenitsa. In: Stylios C., Floqi T., Marinski J., Damiani L. (eds) *Sustainable Development of Sea-Corridors and Coastal Waters*. Springer, Cham. 10.1007/978-3-319-11385-2_22

[CR119] Gross, I., Perez, K., & Quah, B.-L. (2020). *Why Hasn’t Apple Pay Replicated Alipay’s Success?* Harvard Business Review. https://hbr.org/2020/09/why-hasnt-apple-pay-replicated-alipays-success. Accessed 05 May 2021.

[CR120] Gudmundsson SV, Walczuck R (1999). The development of electronic markets in logistics. The International Journal of Logistics Management.

[CR121] Gusenbauer M (2019). Google Scholar to overshadow them all? Comparing the sizes of 12 academic search engines and bibliographic databases. Scientometrics.

[CR122] Gustafsson I (2007). Interaction between transport, infrastructure, and institutional management—Case study of a port community system. Transportation Research Record.

[CR123] Hafsi, N., & Benhayoun, J. (2017). *PortNet in Morocco: Creating a Strategic Alliance between Port and Foreign Trade Communities for a Competitive Economic Operator* (SmartLessons). International Finance Corporation - World Bank Group. https://openknowledge.worldbank.org/bitstream/handle/10986/26294/113167-BRI-IFC-SMART-LESSONS-BRIEF-PUBLIC-20170302T123351–2017-PortNet-in-Morocco-B.pdf. Accessed 19 Mar 2021.

[CR124] Hagiu, A., & Wright, J. (2015). Multi-sided platforms. *International Journal of Industrial Organization,**43*, 162–174.

[CR125] Halevi G, Moed H, Bar-Ilan J (2017). Suitability of Google Scholar as a source of scientific information and as a source of data for scientific evaluation—Review of the Literature. Journal of Informetrics.

[CR126] Hang Seng Bank. (2020). *Hang Seng and OnePort Cross-Sector Collaboration Significantly Reduces Payment Processing Times of e-Orders Reinforces HK’s Position as Key Regional Container Port*. https://www.hangseng.com/cms/ccd/eng/PDF/022420e.pdf. Accessed 15 Mar 2021.

[CR127] Hartmann PM, Zaki M, Feldmann N, Neely A (2016). Capturing value from big data – a taxonomy of data-driven business models used by start-up firms. International Journal of Operations & Production Management.

[CR128] Heathrow Airport. (2017). *Heathrow CargoCloud*. https://www.heathrow.com/content/dam/heathrow/web/common/documents/company/cargo/cargo-cloud/heathrow-cargo-cloud.pdf. Accessed 11 Mar 2021.

[CR129] Hedde-von Westernhagen, C., & Sarigül, S. (2020). 11. Digitalisierung der Hafenarbeit. In S. Möller, T. Gentes, M. Gohlke, J. Jathe, F. Jung, K. Kenanidou, & L. Orlando (Eds.), *Schlüssel zur Welt—Die bremischen Häfen in der Globalen Politischen Ökonomie* (p. 51). https://www.uni-bremen.de/fileadmin/user_upload/fachbereiche/fb8/ipw/Working_Paper/IPW_WorkingPaper_Vol1_Final.pdf. Accessed 15 Apr 2021.

[CR130] Heilig L, Voß S (2017). Information systems in seaports: A categorization and overview. Information Technology and Management.

[CR131] HIT. (2003). *OnePort to Strengthen Hong Kong’s Competitive Edge. *Press Release Hutchison Ports. https://www.hit.com.hk/en/Media-Centre/Press-Release/Oneport.html. Accessed 13 Feb 2021.

[CR132] Hoang, I. S., Herbes, M., Vuong, K. A., & Mai, T. (2014). *Behandlung des Containermanagements in der Logistikliteratur: Vergleich der Lösungsmöglichkeiten Off-Dock Empty Return Depot, Inland Depot for Empty Container, Virtual Container Yard und Full-Service Hinterland-Terminal für Leercontainer.* Schriftenreihe des Lehrstuhls Für ABWL Und Logistikmanagement. https://media.suub.uni-bremen.de/bitstream/elib/3201/1/00105051-1.pdf

[CR133] Hodapp, D., Remane, G., Hanelt, A., & Kolbe, L. M. (2019). Business models for Internet of Things platforms: Empirical development of a taxonomy and archetypes. *14th International Conference on Wirtschaftsinformatik*, February 24–27, 2019, Siegen, Germany.

[CR134] Hong Kong Shippers’ Council. (2005). OnePort launches electronic terminal receipt service. *Shippers Today, 28*(4). http://info.hktdc.com/shippers/vol28_4/vol28_4_ecom_01.htm. Accessed 12 Mar 2021.

[CR135] HPCS. (2021). *Hellenic PCS*. https://hpcs.com.gr/en/ypiresies/. Accessed 10 Mar 2021.

[CR136] HSN. (2020). *CNS introduces new digital Brexit solution to keep hauliers on the move from January | Hellenic Shipping News Worldwide*. Hellenic Shipping News https://www.hellenicshippingnews.com/cns-introduces-new-digital-brexit-solution-to-keep-hauliers-on-the-move-from-january/. Accessed 10 Mar 2021.

[CR137] Hulstijn, J., Hofman, W., Zomer, G., & Tan, Y.-H. (2016). Towards Trusted Trade-Lanes. In: Scholl H. et al. (eds) *Electronic Government. EGOV 2016. Lecture Notes in Computer Science*, vol 9820. Springer, Cham. 10.1007/978-3-319-44421-5_24

[CR138] Hutchinson Whampoa Limited. (2003). *Hutchison Whampoa Limited—Media Center > Press Releases*. http://www.hutchison-whampoa.com/en/media/press_each.php?id=1135. Accessed 11 Mar 2021.

[CR139] Huynh N, Smith D, Harder F (2016). Truck Appointment Systems: Where We Are and Where to Go from Here. Transportation Research Record.

[CR140] IAPH. (2019). *MPCS Project*. IAPH. https://sustainableworldports.org/wp-content/uploads/IAPH-mPCS-Project-2019-.pdf. Accessed 13 Mar 2021.

[CR141] ILCS. (2018). *ILCS Product Portfolio*. https://www.youtube.com/watch?v=v6pjVpBtZNs. Accessed 13 Mar 2021.

[CR142] ILCS. (2020). *ILCS - MY CARGO* https://www.ilcs.co.id/main/page/my-cargo. Accessed 13 Mar 2021.

[CR143] Indian Ports Association. (2020). *Design, development, integration, implementation, operation and maintenance of National Logistics Portal (Marine)*. https://vizagport.com/wp-content/uploads/2020/10/NLPRFPVol2.pdf. Accessed 15 Mar 2021.

[CR144] Interreg. (2019). *RPIS 4.0 – Smart Community System for Upper Rhine Ports*. https://www.interreg-oberrhein.eu/projet/rpis-4–0-smart-community-system-for-upper-rhine-ports/. Accessed 13 Mar 2021.

[CR145] IPCSA. (2017). *Dubai Trade Case Study*. https://ipcsa.international/armoury/resources/ipcsacasestudydubaifinal.pdf. Accessed 10 Mar 2021.

[CR146] IPCSA. (2020). *Network of Trusted Networks – IPCSA International*. https://ipcsa.international/initiatives/network-of-trusted-networks/. Accessed 10 Mar 2021.

[CR147] Israel Port Authority. (2020). *About Israel Port Community System*. http://www.israports.co.il/en/ipcs/Pages/default.aspx. Accessed 13 Mar 2021.

[CR148] Israel Ports. (2020). *Israel Ports Company has begun an innovative pilot for transferring bills of lading, using blockchain technology*. https://israports.co.il/en/IPCS/Documents/IPCSA%20BOL%20BLOCKCHAIN%20INITIATIVE.pdf. Accessed 09 Mar 2021.

[CR149] ITJ. (2015). *MGI: Ci5 instead of AP+.* International Transport Journal*.* https://www.transportjournal.com/en/home/news/artikeldetail/mgi-ci5-instead-of-ap.html. Accessed 15 Mar 2021.

[CR150] Jackson. (2016). *Roll out of Port Community System under way*. http://jamaica-gleaner.com/article/business/20160713/roll-out-port-community-system-under-way. Accessed 15 Mar 2021.

[CR151] Jain, A. K., Duin, R. P. W., & Mao, J. (2000). Statistical pattern recognition: A review. *IEEE Transactions on Pattern Analysis and Machine Intelligence,**22*(1), 4–37. 10.1109/34.824819

[CR152] Jaklitsch, M. (2013). *Deutliches Umsatzplus bei dbh Logistics IT AG.* LOGISTIK Express NEWS. https://www.logistik-express.com/deutliches-umsatzplus-bei-dbh-logistics-it-ag/. Accessed 08 Mar 2021.

[CR153] J.M. Baxi Group (2019). Implementing The IPA PCS1x In INDIA An Honour And Privilege For PORTALL. TIDINGS.

[CR154] Joszczuk–Januszewska, J. (2012). The benefits of cloud computing in the maritime transport. In J. Mikulski (Ed.), *Telematics in the transport environment, *Vol. 329, pp. 258–266. Springer, Berlin Heidelberg. 10.1007/978-3-642-34050-5_29

[CR155] Jouad S, Hamri MH (2020). The impact of information systems on port performance: The case of Morocco’s Agadir Port. European Scientific Journal.

[CR156] Jović, M., Tijan, E., Aksentijević, S., & Čišić, D. (2019). An Overview Of Security Challenges Of Seaport IoT Systems. *42nd International Convention on Information and Communication Technology, Electronics and Microelectronics (MIPRO)*, 1349–1354. 10.23919/MIPRO.2019.8757206

[CR157] Kale Logistics. (2019). *North America’s first New Generation Airport Cargo Community Systemdeveloped by Kale in partnership with Atlanta Air freight community and Atlanta airport goes live at Hartsfield Jackson International Airport*. https://www.kalelogistics.com/north-americas-first-new-generation-airport-cargo-community-system/. Accessed 06 Mar 2021.

[CR158] Kale Logistics. (2020a). *Codex Port Community System in India*. https://www.kalelogistics.com/trade-facilitation/pcs/. Accessed 11 Mar 2021.

[CR159] Kale Logistics. (2020b). *Creating North America’s First Air Cargo Community System*. https://www.kalelogistics.com/creating-north-americas-first-air-cargo-community-system/. Accessed 10 Mar 2021.

[CR160] Kamel, D. (2020). *Abu Dhabi Ports acquires Micco Logistics as it targets underserved markets*. The National News. https://www.thenationalnews.com/business/economy/abu-dhabi-ports-acquires-micco-logistics-as-it-targets-underserved-markets-1.1084197. Accessed 15 Mar 2021.

[CR161] Kapkaeva N, Gurzhiy A, Maydanova S, Levina A (2021). Digital platform for maritime port ecosystem: Port of Hamburg case. Transportation Research Procedia.

[CR162] Kelle U (2006). Combining qualitative and quantitative methods in research practice: Purposes and advantages. Qualitative Research in Psychology.

[CR163] Kenyon GN, Goldsmith M, Neureuther BD, Zhou D (2018). Improving the return on investment in ports: Opportunities in data management. Maritime Economics & Logistics.

[CR164] Keretho, S., & Pikart, M. (2013). *Trends for collaboration in international trade: Building a common Single Window Environment* (ECE-TRADE 411, p. 43). UNECE. https://www.unece.org/ece/trade/411.html. Accessed 11 Mar 2021.

[CR165] KIZAD. (2020). *KIZAD Rolls Out New Set of Digital Services through Maqta Gateway*. Khalifa Industrial Zone Abu Dhabi (KIZAD). https://www.kizad.ae/2020/04/27/kizad-rolls-out-new-set-of-digital-services-through-maqta-gateway/. Accessed 19 Mar 2021.

[CR166] Klijn, E.-H., & Teisman, G. R. (2003). Institutional and strategic barriers to public—private partnership: An analysis of Dutch cases. *Public Money and Management,**23*(3), 137–146. 10.1111/1467-9302.00361

[CR167] KLNET. (2020). *PLISM3.0*. https://www.plism.com/websquare/websquare.html?w2xPath=/sq5/com/uat/svc/Start.xml&subPage=/sq5/com/uat/svc/SvcChargeGuide.xml&topMenuNo=1000. Accessed 21 Mar 2021.

[CR168] Kumar, R. (2019). *Lessons to learn from the failure of GE’s IoT Platform, Predix?* Medium. https://medium.com/world-of-iot/73-lessons-to-learn-from-the-failure-of-ges-iot-platform-predix-3b3d20eccd42. Accessed 18 Mar 2021.

[CR169] Leach, P. T. (2012). *Indonesia to implement SOGET-Microsoft Port Community System.* JOC. https://www.joc.com/port-news/indonesia-implement-soget-microsoft-port-community-system_20120927.html. Accessed 16 May 2021.

[CR170] Lee KJ, Simpson JA (2014). Introduction to multiple imputation for dealing with missing data. Respirology.

[CR171] Lee T-W, Park N-K, Joint JF, Kim WG (2000). A new efficient EDI system for container cargo logistics. Maritime Policy & Management.

[CR172] Lee-Partridge JE, Teo TSH, Lim VKG (2000). Information technology management: The case of the Port of Singapore Authority. The Journal of Strategic Information Systems.

[CR173] Leviäkangas, P., & Hautala, R. (2002). Impact evaluation of maritime ITS–Case PortNet. *The World Congress on ITS,**9*, 14–17. http://virtual.vtt.fi/virtual/proj6/fits/impacts/PortNet_evaluation_paper_final.pdf

[CR174] Li, F. (2019). Why have all western internet firms (WIFs) failed in China? A phenomenon-based study. *Academy of Management Discoveries,**5*(1), 13–37. 10.5465/amd.2017.0102

[CR175] Li, H., & Hecht, B. (2021). 3 stars on Yelp, 4 stars on Google Maps: A cross-platform examination of restaurant ratings. *Proceedings of the ACM on Human-Computer Interaction,**4*(CSCW3), 1–25. 10.1145/3432953

[CR176] Lievens, D. (2017). *NxtPort - The Next Level. Presentation. *https://www.nxtport.com/media/presentations/20171212_nxtport_thenextlevel.pdf. Accessed 09 Dec 2020.

[CR177] Linton, L. (2020). *Port community system making doing business easier during COVID-19. *Jamaica Information Service. https://jis.gov.jm/features/port-community-system-making-doing-business-easier-during-covid-19/. Accessed 10 Mar 2021.

[CR178] Long, A. (2009). Port community systems. *World Customs Journal,**3*(1), 63–69. 10.1016/j.tra.2019.12.021

[CR179] Loux P, Aubry M, Tran S, Baudoin E (2020). Multi-sided platforms in B2B contexts: The role of affiliation costs and interdependencies in adoption decisions. Industrial Marketing Management.

[CR180] Lu Y-C, Xiao Y, Sears A, Jacko JA (2005). A review and a framework of handheld computer adoption in healthcare. International Journal of Medical Informatics.

[CR181] MACCS. (2017). *CCS Fee applicable on Import of Consignments /Goods by Pos*. https://www.maccs.mu/maccs/wp-content/uploads/2017/01/MaCCS_-Communique_Go-Live-_Submission-of-Postal-Manifest-through-CCS-_v4_16012017.pdf. Accessed 22 Mar 2021.

[CR182] MACCS. (2020). *MACCS – History & Milestones*. https://www.maccs.mu/history-milestones/. Accessed 21 Mar 2021.

[CR183] Madley-Dowd P, Hughes R, Tilling K, Heron J (2019). The proportion of missing data should not be used to guide decisions on multiple imputation. Journal of Clinical Epidemiology.

[CR184] Marek R, Dujak D (2017). The role and place of customs in port community system—experiences from Poland. Business logistics in modern management.

[CR185] Margo. (2020). *Margo | From Port or Airport to Your Doorstep*. https://margo.ae/. Accessed 22 Mar 2021.

[CR186] Marin Portillo A, Basterrechea Iribar I (2020). Study of the procedures and telematic means for the entry of a vessel into the port.

[CR187] Maritime Gateway. (2020a). *Customs API goes live on PCS1X*. http://www.maritimegateway.com/customs-api-goes-live-pcs1x/. Accessed 11 Mar 2021.

[CR188] Maritime Gateway. (2020b). *Freight booking now via PCS 1x: BoxnBiz Collaborates With P- CaSo.* http://www.maritimegateway.com/freight-booking-now-via-pcs-1x-boxnbiz-collaborates-p-caso/. Accessed 10 Mar 2021.

[CR189] Maritime Gateway. (2020c). *Custodians spur the API integrations with PCS 1x*. https://www.maritimegateway.com/custodians-spur-api-integrations-pcs-1x/. Accessed 01 Mar 2021.

[CR190] Martín-Martín A, Thelwall M, Orduna-Malea E, Delgado López-Cózar E (2021). Google Scholar, Microsoft Academic, Scopus, Dimensions, Web of Science, and OpenCitations’ COCI: A multidisciplinary comparison of coverage via citations. Scientometrics.

[CR191] Matczak, M. (2013). Intelligent container terminals-ITS solutions for seaports. *Archives of Transport SystemTelematics*, 6(2), 35–40.

[CR192] Mathew, B. (2019). *International-Logistics: India eyes global integration for digital port platform.* JoC Online. https://www.joc.com/international-logistics/logistics-technology/india-eyes-global-links-digital-port-platform_20191001.html. Accessed 15 Mar 2021.

[CR193] Mayanti B, Kantola J, Natali M, Kytola J, Carpenter A, Lozano R (2020). Analysing port community system network evolution. European port cities in transition: Moving towards more sustainable sea transport hubs.

[CR194] McCrum-Gardner, E. (2008). Which is the correct statistical test to use? *British Journal of Oral and Maxillofacial Surgery,**46*(1), 38–41. 10.1016/j.bjoms.2007.09.002 10.1016/j.bjoms.2007.09.002 17961892

[CR195] MCP. (2020). *Destin8*. https://www.mcpplc.com/Products-&-Services/Destin8.aspx. Accessed 20 Dec 2020.

[CR196] MGI. (2020a). *Ci5*. Google Play Store. https://play.google.com/store/apps/details?id=com.mgi.ci5&hl=de&gl=DE. Accessed 20 Dec 2020.

[CR197] MGI. (2020b). *MGI - Ci5 Core—Takes us into the Smart Port Area*. https://www.mgi-ci5.com/en/ci5-core/. Accessed 20 Dec 2020.

[CR198] MGI. (2020c). *MGI - Ci5—Discover the Cargo Intelligent System.*. https://www.mgi-ci5.com/en/ci5/. Accessed 20 Dec 2020.

[CR199] Mongelluzzo, B. (2018a). *Port of Los Angeles Port Optimizer: US port portals enter new markets with new features.* JoC Online. https://www.joc.com/port-news/us-ports/port-los-angeles/us-port-portals-enter-new-markets-new-features_2018a0816.html. Accessed 20 Feb 2021.

[CR200] Mongelluzzo, B. (2018b). *Port of Los Angeles information portal: Fluidity-boosting LA portal grapples with public-private issue*. JoC Online. https://www.joc.com/port-news/us-ports/port-los-angeles/incentives-la-port-portal-boost-fluidity-raises-public-private-issue_2018b0827.html. Accessed 20 Feb 2021.

[CR201] Moros-Daza A, Amaya-Mier R, Paternina-Arboleda C (2020). Port community systems: A structured literature review. Transportation Research Part a: Policy and Practice.

[CR202] Moros-Daza, A. M., Amaya, R. A., Garcia, G., & Paternina, C. (2016). Assessing the effect of implementing a port community system platform in the response time of an international terminal: The case of a multi- cargo facility at the Colombian Caribbean coast. *2016 International Conference on Industrial Engineering and Operations Management (IEOM), IEEE Xplore*, 10.

[CR203] Morton, R. (2015). *IPCSA - How to developa Port Community System*. International Port Community Systems Association. http://www.ipcsa.international/armoury/resources/ipcsa-guide-english-2015.pdf. Accessed 11 Dec 2020.

[CR204] Morton, R. (2018). *The Development of Port Community Systems (PCS) and integration with Single Window* [IPCSA]. https://www.sicexchile.cl/portal/documents/10180/13179/IPCSA_presentation.pdf/30f00211-91c4-4dc3-9f5c-997eda99a190. Accessed 11 Dec 2020.

[CR205] Moyersoen L (2019). Building the Port of the Future.

[CR206] Nallian. (2021a). *DFW Cargo Cloud*. https://www.nallian.com/communities/dfw-cargo-cloud. Accessed 03 Mar 2021.

[CR207] Nallian. (2021b). *Heathrow Cargo Cloud*. https://www.nallian.com/communities/heathrow-cargo-cloud. Accessed 03 Mar 2021.

[CR208] Nallian. (2021c). *Liege Cargo Cloud | Nallian*. https://www.nallian.com/communities/lgg-cargo-cloud. Accessed 03 Mar 2021.

[CR209] Nallian. (2021d). *Lux-Airport*. https://www.nallian.com/communities/lux-airport. Accessed 03 Mar 2021.

[CR210] Nallian. (2021e). *Nallian*. Home. https://www.nallian.com/. Accessed 03 Mar 2021.

[CR211] Nallian. (2021f). *Vienna Cargo Cloud*. https://www.nallian.com/communities/vie-cargo-cloud. Accessed 03 Mar 2021.

[CR212] Nathan Associates. (2013). *Logistics Cost Study of Transport Corridors in Central and West Africa* (World Bank - Africa Transport Unit No. 7161353). World Bank. https://www.ssatp.org/sites/ssatp/files/publications/SSATP_Logistics_Cost_Study_Complete%20with%20annexes%20Final%20September%202013.pdf. Accessed 12 Mar 2021.

[CR213] Nemeth, C., Brown, K., & Rogers, J. (2001). Devil’s advocate versus authentic dissent: Stimulating quantity and quality. *European Journal of Social Psychology,**31*(6), 707–720. 10.1002/ejsp.58

[CR214] Neo BS (1994). Managing new information technologies: Lessons from Singapore’s experience with EDI. Information & Management.

[CR215] Neo B-S, Khoo P, Green C (1994). The adoption of TradeNet by the trading community: An empirical analysis. ICIS 1994 Proceedings.

[CR216] Ng Cheong Hin, D. (2013). Deployment and Financing of the Port Community System in Mauritius. *AACE International Single Window Conference & Exhibition*, Antananarivo, Madagascar. http://alliance-africaine.org/SWC2013/fr/presentation/aace_1.pdf. Accessed 03 Mar 2021.

[CR217] Nickerson RC, Varshney U, Muntermann J (2013). A method for taxonomy development and its application in information systems. European Journal of Information Systems.

[CR218] NTP. (2020). *Overview; Government VAS ; Value-Added Services (VAS)*. Networked Trade Platform - Overview. https://www.ntp.gov.sg/public/introduction-to-ntp---overview; https://www.ntp.gov.sg/public/browse-govvas-catalogue; https://www.ntp.gov.sg/public/browse-vas-catalogue. Accessed 22 Dec 2020.

[CR219] NxtPort. (2018). *Towards a competitive advantage*. European Freight Leaders Forum. https://www.europeanfreightleaders.eu/wp-content/uploads/2018/02/305.-NxtPort-intiative.pdf. Accessed 22 Dec 2020.

[CR220] NxtPort. (2020a). *Information Security Policy*. https://www.nxtport.com/nxtport/itsecurity. Accessed 22 Dec 2020.

[CR221] NxtPort. (2020b). *Logit One—Visibility tool*. https://www.nxtport.com/market/applications/logit-one. Accessed 22 Dec 2020.

[CR222] OnePort. (2020). *OnePort Limited*. OnePort - Our Services. https://www.oneport.com/eng/our_services.htm. Accessed 22 Dec 2020.

[CR223] OnePort Limited. (2014). OnePort. *Seminar on APEC Model E-Ports,* Beijing, China. http://mddb.apec.org/Documents/2014/CTI/SEM1/14_cti_sem1_006.pdf. Accessed 12 Dec 2020.

[CR224] O’reilly T (2007). What is Web 2.0: Design patterns and business models for the next generation of software. Communications & Strategies.

[CR225] Osei-Owusu, J. Y., & Mahmood, R. (2020). Port paperless system in Ghana, the way forward: GCNET or UNIPASS/ICUMS. *ADRRI Journal of Arts and Social Sciences,**17*(6 (5)), 72–87. 10.55058/adrrijass.v17i6(5).544

[CR226] Osterwalder A, Pigneur Y (2010). Business model generation: A handbook for visionaries, game changers, and challengers.

[CR227] Parker GG, Van Alstyne MW, Choudary SP (2016). Platform revolution: How networked markets are transforming the economy and how to make them work for you.

[CR228] Passlick, J., Dreyer, S., Olivotti, D., Grützner, L., Eilers, D., & Breitner, M. H. (2021). Predictive maintenance as an internet of things enabled business model: A taxonomy. *Electronic Markets, 31*(1). 10.1007/s12525-020-00440-5

[CR229] Patwardhan, R. (2018, March 28). *Kale Logistics Solutionslaunches RIGEL,India’s first online e-Booking platformforthe air cargo industryat WINGS 2018*. https://theloadstar.com/wp-content/uploads/PR_Kale-launches-e-booking-platform.pdf. Accessed 24 Dec 2020.

[CR230] PayCargo. (2020). *PayCargo collaborates with Atlanta Airport and Kale Logistics to launch first air cargo community system in the USA*. Meantime Communications. https://meantime.global/news/paycargo-collaborate-with-atlanta-airport-and-kale-logistics-to-launch-first-air-cargo-community-system-in-the-usa/. Accessed 25 Dec 2020.

[CR231] PCS e-port. (2008). *E-Port overview*. http://www.pcs-eport.it/eportHomePage/documents/brochureEport2008ENG.pdf. Accessed 22 Dec 2020.

[CR232] Peng C-C, Tsai C-J, Chang T-Y, Yeh J-Y, Hua P-W (2020). A new approach to generate diversified clusters for small data sets. Applied Soft Computing.

[CR233] Pereira, B. (2020). *Kale Logistics Solutions deploys CODEX for V. O. Chidambaranar Port Trust.* Digital Creed. https://www.digitalcreed.in/kale-logistics-solutions-deploys-codex/. Accessed 15 Dec 2020.

[CR234] Petrakakos, N. H. (2005). *Port security and information technology *(Thesis). Massachusetts Institute of Technology. https://dspace.mit.edu/handle/1721.1/33573. Accessed 20 Mar 2021.

[CR235] PMAC. (2017). *Port Management Association of the Caribbean—SOGET: 10 YEAR International track Record! *http://www.pmac-ports.com/news/99-soget-10-year-international-track-record.Accessed 02 Dec 2020.

[CR236] Polydoropoulou, A., Lambrou, M. A., Roumboutsos, A., & Kourounioti, I. (2011). Investigating the factors affecting the successful implementation of a port community system. *Proceedings of the European Conference on Shipping & Ports (ECONSHIP),* 14.

[CR237] PORT AUTHORITY OF IGOUMENITSA. (2014). *Functional Specification Requirementsof The “APC” Project*. http://olig.gr/sites/default/files/APC%20FUNCTIONAL%20SPECIFICATION%20REQUIREMENTS%20OLIG%20SA.pdf. Accessed 25 Dec 2020.

[CR238] Port Authority of Savonna. (2021). *Benvenuto in PCS - Port Community System Savona*. http://pcs.porto.sv.it/. Accessed 22 Dec 2021.

[CR239] Port of Antwerp. (2020). *Electronic solutions for a clearer supply chain*. Port of Antwerp. http://www.portofantwerp.com/en/node/14630. Accessed 22 Dec 2020.

[CR240] Port of Los Angeles. (2020). *Port Optimizer*^*TM*^* | Supply Chain | Port of Los Angeles*. https://www.portoflosangeles.org/business/supply-chain/port-optimizer%e2%84%a2. Accessed 22 Dec 2020.

[CR241] Port of New York and New Jersey. (2021). *Community Portal*. https://www.porttruckpass.com/. Accessed 22 Mar 2021.

[CR242] Port Strategy. (2017). *Blockchain alliance battles fake products*. https://www.portstrategy.com/news101/products-and-services/blockchain-alliance-battles-fake-products. Accessed 22 Dec 2020.

[CR243] Port Technology. (2020). *Port of Gothenburg and Wabtec to implement Port Optimizer*. Port Technology International. https://www.porttechnology.org/news/port-of-gothenburg-and-wabtec-to-implement-port-optimizer/. Accessed 22 Dec 2020.

[CR244] Portall. (2020). *Portall PCS - PCS 1x*. Portall Offerings. https://www.portall.in/offerings. Accessed 22 Dec 2020.

[CR245] Portbase. (2020). *How it works; Services; Portbase developer portal; “Through collaboration with Portbase, our software is developing fast"*. https://www.portbase.com/en/port-community-system/; https://www.portbase.com/services/; https://developer.portbase.com/; https://www.portbase.com/en/klantervaringen/through-collaboration-with-portbase-our-software-is-developing-fast/. Accessed 12 Dec 2020.

[CR246] Portic. (2020). *Services: Forwarding Agent & Customs Agent -- Freight Haulier -- Shipping Agent*. http://www.portic.net/ENG/ficha_transitario2.html --http://www.portic.net/ENG/ficha_transportista2.html -- http://www.portic.net/ENG/ficha_consignatario2.html. Accessed 22 Dec 2020.

[CR247] Portic Barcelona. (2015). *Portic—Connecting The Port Community"*. http://www.portic.net/ENG/crecimento_expo.shtml. Accessed 02 Jan 2021.

[CR248] PORTNET. (2020). *Our security policy*. https://www.portnet.ma/en/notre-politique-de-securite. Accessed 24 Dec 2020.

[CR249] Ports of Sines and the Algarve Authority, S. A. (2020). *JUPII/JUL - Logistic Single Window*. http://www.apsinesalgarve.pt/en/it-systems/jupiijul-logistic-single-window/. Accessed 25 Dec 2020.

[CR250] Posti A, Häkkinen J, Tapaninen U, Kersten W, Blecker T, Jahn C (2011). Promoting information exchange with a port community system–case Finland. International supply chain management and collaboration practices.

[CR251] Powell, D. (2017). *AN analysis of the port community system implementation in Jamaica*. Regional Latin American & Caribbean Meeting of PORT LOGISTICS. http://s017.sela.org/media/2465199/bloque-2-2-jamaica-pcs.pdf. Accessed 28 Dec 2020.

[CR252] Putzger, I. (2020a). *Dallas/Fort Worth gets green light for cargo community system to take off.* The Loadstar. https://theloadstar.com/dallas-fort-worth-gets-green-light-for-cargo-community-system-to-take-off/. Accessed 22 Dec 2020.

[CR253] Putzger, I. (2020b). *Schiphol’s cargo community readies for vaccines storm*. The Loadstar. https://theloadstar.com/schiphols-cargo-community-readies-for-vaccines-storm/. Accessed 22 Dec 2020.

[CR254] Reck, J. (2021).* The regulation of tech monopolies will decide the fate of Western democracies*. Business Insider. https://www.businessinsider.com/regulation-of-big-tech-decide-fate-of-western-democracies-2021–2. Accessed 25 Mar 2021.

[CR255] Remane G, Hanelt A, Nickerson RC, Kolbe LM (2017). Discovering digital business models in traditional industries. Journal of Business Strategy.

[CR256] Remane, G., Nickerson, R., Hanelt, A., Tesch, J. F., & Kolbe, L. M. (2016). A taxonomy of carsharing business models. *ICIS 2016 Proceedings.* https://aisel.aisnet.org/icis2016/Crowdsourcing/Presentations/18. Accessed 05 Apr 2021.

[CR257] Reuters. (2020). *UPDATE 1-Greece gets strong non-binding interest for Igoumenitsa port*. https://www.reuters.com/article/greece-privatisations-igoumenitsa-idUSL8N2HL784. Accessed 22 Dec 2020.

[CR258] Riemensperger F, Falk S (2020). How to capture the B2B platform opportunity. Electronic Markets.

[CR259] Robey D, Im G, Wareham JD (2008). Theoretical foundations of empirical research on interorganizational systems: Assessing past contributions and guiding future directions. Journal of the Association for Information Systems.

[CR260] Rodon J, Pastor JA, Sese F, Christiaanse E (2008). Unravelling the dynamics of IOIS implementation: An actor-network study of an IOIS in the seaport of Barcelona. Journal of Information Technology.

[CR261] Rodon, J., & Ramis-Pujol, J. (2006). Exploring the intricacies of integrating with a port community system. *BLED 2006 Proceedings*, 9. https://aisel.aisnet.org/bled2006/9. Accessed 22 Dec 2020.

[CR262] Rodon J, Ramis-Pujol J, Christiaanse E (2007). A process-stakeholder analysis of B2B industry standardisation. Journal of Enterprise Information Management.

[CR263] Roychowdhury, I. (2019). *How Port Community System, an e-commerce portal, is changing India’s maritime infrastructure*. The Financial Express. https://www.financialexpress.com/infrastructure/how-port-community-system-an-e-commerce-portal-is-changing-indias-maritime-infrastructure/1685933/. Accessed 04 Dec 2020.

[CR264] Rukanova, B., Henningsson, S., Henriksen, H. Z., & Tan, Y.-H. (2018). Digital trade infrastructures: A framework for analysis. *Complex Systems Informatics and Modeling Quarterly,**14*, 1–21. 10.1007/s12525-019-00352-z

[CR265] Saebi, T., Lien, L., & Foss, N. J. (2017). What drives business model adaptation? The impact of opportunities, threats and strategic orientation. *Long Range Planning,**50*(5), 567–581. 10.1016/j.lrp.2016.06.006

[CR266] Saeed I, Juell-Skielse G, Uppström E (2012). Cloud enterprise resource planning adoption: Motives & barriers. Advances in Enterprise Information Systems I.

[CR267] Salvador, S., & Chan, P. (2004). Determining the number of clusters/segments in hierarchical clustering/segmentation algorithms. *16th IEEE International Conference on Tools with Artificial Intelligence*, 576–584. 10.1109/ICTAI.2004.50

[CR268] Sathasivam K (2009). The single electronic window – Singapore’s TradeNet – scope of services and pricing model.

[CR269] Schafer JL, Olsen MK (1998). Multiple imputation for multivariate missing-data problems: A data analyst’s perspective. Multivariate Behavioral Research.

[CR270] Schoeters, M. (2016a).* BRU Airport’s Cloud Platform is Taking Shape—PART 1*. CargoForwarder Global. http://www.cargoforwarder.eu/2016a/07/18/bru-airport-s-cloud-platform-is-taking-shape-part-1/. Accessed 11 Dec 2020.

[CR271] Schoeters, M. (2016b). *BRU Airport’s Cloud Platform—PART 2*. CargoForwarder Global. http://www.cargoforwarder.eu/2016b/07/24/part-2-bru-airport-s-cloud-platform/. Accessed 08 Dec 2020.

[CR272] Schoormann, T., Behrens, D., Kolek, E., & Knackstedt, R. (2016). Sustainability in business models – A literature-review-based design-science-oriented research agenda. *Proceedings of the 24th European Conference on Information Systems (ECIS 2016). Istanbul, Turkey*. https://aisel.aisnet.org/ecis2016_rp/134. Accessed 01 Dec 2020.

[CR273] Shaikh, A. (2018). *Blockchain technology to drive port community system by December*. DNA. http://search.ebscohost.com/login.aspx?direct=true&db=bsu&AN=131983097&site=ehost-live. Accessed 09 Dec 2020.

[CR274] Silcock, R. (2001). What is e-government. *Parliamentary Affairs,**54*(1), 88–101. 10.1093/pa/54.1.88

[CR275] Simoni, M., Schiavone, F., Risitano, M., Leone, D., & Chen, J. (2020). Group-specific business process improvements via a port community system: The case of Rotterdam. *Production Planning & Control*, 1–15. 10.1080/09537287.2020.1824029

[CR276] Simushkov, A., & Korovyakovsky, E. (2008). Principles of formation of transport and logistic complex common information area. *Fourth International Railway Logistics Seminar: Co-Operation among Transportation Modes in Northern Europe*, 147–162. http://ek0.ru/LUT/200_fourth_railway_forum.pdf#page=150. Accessed 21 Mar 2021.

[CR277] Sinfomar. (2015). *Sinfomar—Port community system*. https://www.sinfomar.it/wp-content/uploads/2015/02/Sinfomar-presentazione-2015.pdf. Accessed 11 Dec 2020.

[CR278] Singapore Customs. (2018). *Going beyond the national Single Window*. WCO News. https://mag.wcoomd.org/magazine/wco-news-87/going-beyond-the-single-window/. Accessed 22 Nov 2020.

[CR279] Singapore Customs. (2020). *Leaning forward: Singapore leverages digital data to help financial institutions augment trade finance compliance. *WCO News 92. https://mag.wcoomd.org/magazine/wco-news-92-june-2020/leaning-forward-singapore-leverages-digital-data-to-help-financial-institutions-augment-trade-finance-compliance/. Accessed 10 Dec 2020.

[CR280] Smith, J. (2019). *Schiphol airport eyes closer supply chain cooperation with Dutch government.* FreightWaves. https://www.freightwaves.com/news/schiphol-airport-eyes-closer-supply-chain-cooperation-with-dutch-government. Accessed 08 Dec 2020.

[CR281] SOGET. (2011). *Cotonou’s Port Single Window official Go-live*. http://www.soget.fr/en/soget-siege-3/news/item/cotonou-s-port-community-system-official-go-live.html. Accessed 21 Dec 2020.

[CR282] SOGET. (2019). *SOGET digital platforms handle more than half a million secured messages a day.* Hellenic Shipping News Worldwide. https://www.hellenicshippingnews.com/soget-digital-platforms-handle-more-than-half-a-million-secured-messages-a-day/. Accessed 20 Dec 2020.

[CR283] SOGET. (2020a). *Benin Port Community System*. https://www.soget.fr/en/customers/cotonou.html. Accessed 22 Dec 2020.

[CR284] SOGET. (2020b). *DR of Congo Port Community System*. https://www.soget.fr/en/customers/d-r-of-the-congo.html. Accessed 22 Dec 2020.

[CR285] SOGET. (2020c). *Indonesia Port Community System*. https://www.soget.fr/en/customers/jakarta.html. Accessed 22 Dec 2020.

[CR286] SOGET. (2020d). *Jamaica Port Community System*. https://www.soget.fr/en/customers/jamaica.html. Accessed 22 Dec 2020.

[CR287] SOGET. (2020e). *Le Havre Port Community System*. https://www.soget.fr/en/customers/france/le-havre-uk.html. Accessed 22 Dec 2020.

[CR288] SOGET. (2020f). *Mauritius Port Community System*. http://www.sogetnews.fr/en/customers/jamaica/12-communautes-portuaires/88-implementation-of-port-single-window-in-port-louis.html. Accessed 22 Dec 2020.

[CR289] SOGET. (2020g). *Togo Port Community System*. https://www.soget.fr/en/customers/lome.html. Accessed 22 Dec 2020.

[CR290] SOGET. (2021). *Guadeloupe Port Community System*. https://www.soget.fr/en/customers/france/pointe-a-pitre.html. Accessed 12 Mar 2021.

[CR291] Sokal RR, Michener CD (1958). A statistical method for evaluating systematic relationships. University of Kansas Science Bulletin.

[CR292] Srour, F. J., van Oosterhout, M., van Baalen, P., & Zuidwijk, R. (2008). Port community system implementation: Lessons learned from international scan. *Transportation Research Board 87th Annual Meeting, Washington DC*.

[CR293] Stat Times. (2020). *Schiphol Airport to work with digital pre-notifications for export cargo from Jan 2021.*. https://www.stattimes.com/news/schiphol-airport-to-work-with-digital-prenotifications-for-export-cargo-from-jan-2021-aviation/. Accessed 10 Dec 2020.

[CR294] Šulc Z, Řezanková H (2019). Comparison of Similarity Measures for Categorical Data in Hierarchical Clustering. Journal of Classification.

[CR295] Susha, I., Janssen, M., & Verhulst, S. (2017). Data Collaboratives as a New Frontier of Cross-Sector Partnerships in the Age of Open Data: Taxonomy Development. *Hawaii International Conference on System Sciences.*10.24251/HICSS.2017.325

[CR296] Szopinski D, Schoormann T, John T, Knackstedt R, Kundisch D (2020). Software tools for business model innovation: Current state and future challenges. Electronic Markets.

[CR297] Tan, B., Pan, S., Lu, X., & Huang, L. (2015). The Role of IS Capabilities in the Development of Multi-Sided Platforms: The Digital Ecosystem Strategy of Alibaba.com. *Journal of the Association for Information Systems*, *16*(4). 10.17705/1jais.00393

[CR298] Taneja P, Walker WE, Ligteringen H, Schuylenburg MV, Plas RVD (2010). Implications of an uncertain future for port planning. Maritime Policy & Management.

[CR299] Tang, L., Shen, Q., & Cheng, E. W. (2010). A review of studies on public–private partnership projects in the construction industry. *International Journal of Project Management,**28*(7), 683–694. 10.1016/j.ijproman.2009.11.009

[CR300] Täuscher, K., & Laudien, S. M. (2018). Understanding platform business models: A mixed methods study of marketplaces. *European Management Journal,**36*(3), 319–329. 10.1016/j.emj.2017.06.005

[CR301] TaylorWessing. (2020). *2021 predictions: Increased regulation of online platforms*. Insights. https://www.taylorwessing.com/en/insights-and-events/insights/2020/12/2021-predictions-increased-regulation-of-online-platforms. Accessed 22 Dec 2020.

[CR302] Teece DJ (2010). Business Models, Business Strategy and Innovation. Long Range Planning.

[CR303] Teo, H.-H., Tan, B. C. Y., & Wei, K.-K. (1997). Organizational transformation using electronic data interchange: The case of TradeNet in Singapore. *Journal of Management Information Systems,**13*(4), 139–165. 10.1080/07421222.1997.11518146

[CR304] The Business Times. (2018).* Singapore’s new digital trade platform can help build cross-border linkages*. https://www.businesstimes.com.sg/government-economy/singapores-new-digital-trade-platform-can-help-build-cross-border-linkages. Accessed 29 Dec 2020.

[CR305] TREDIT. (2014). *FREightight Transport InformationTechnology Solution*. http://tredit.gr/wp-content/uploads/2014/11/fretis.pdf. Accessed 29 Dec 2020.

[CR306] TREDIT. (2020). *TREDIT’s FRETIS-IFT TOS now interfaced to TSB’s CATOS Ship Planning | TREDIT S.A. *http://tredit.gr/?p=1631&lang=en. Accessed 28 Dec 2020.

[CR307] Tremblay, M. C., Hevner, A. R., & Berndt, D. J. (2010). Focus Groups for Artifact Refinement and Evaluation in Design Research. *Communications of the Association for Information Systems*, *26*. 10.17705/1CAIS.02627

[CR308] Tsamboulas, D., & Ballis, A. (2013). Port Community systems: Requirements, functionalities and implementation complications. *Selected Proceedings of the 13th World Conference of Transport Research, Rio de Janeiro*, 1–16.

[CR309] Tulli. (2020). *Port Traffic Declaration Service (Portnet)*. https://tulli.fi/en/e-services/services/port-traffic-declaration-service-portnet. Accessed 29 Dec 2020.

[CR310] UNECE. (2017). *The use of UN/LOCODE in Cargo Community Systems*. https://unece.org/fileadmin/DAM/cefact/cf_forums/2017_Geneva/PPTs/LOCODE/S3.2-CargoCommunitySystems_Marseille.pdf. Accessed 26 Dec 2020.

[CR311] UNINA, & RAM. (2015). *MED-PCS Promotion of “Port Community System” in mediterranean traffic* (General Study No. D411; Implementation of the PCS in Europe).

[CR312] Vairetti C, González-Ramírez RG, Maldonado S, Álvarez C, Voβ S (2019). Facilitating conditions for successful adoption of inter-organizational information systems in seaports. Transportation Research Part A: Policy & Practice.

[CR313] ValenciaportPCS. (2012). *Official port dues for the use of the services of valenciaportpcs.net*. https://www.valenciaportpcs.com/media/1123/tariffs_valenciaportpcs.pdf. Accessed 12 Dec 2020.

[CR314] ValenciaportPCS. (2020a). *Faqs | ValenciaportPCS*. Frequently Asked Questions. https://www.valenciaportpcs.com/en/support/faqs/. Accessed 22 Dec 2020.

[CR315] ValenciaportPCS. (2020b). *Transporte Terrestre valenciaportPCS*. Google Play Store. https://play.google.com/store/apps/details?id=net.valenciaportpcs.transport&hl=de&gl=DE. Accessed 22 Dec 2020.

[CR316] Van Baalen, P., Zuidwijk, R., & Van Nunen, J. (2009). *Port inter-organizational information systems**: **Capabilities to service global supply chains* (Vol. 2). 10.1561/0200000008

[CR317] Van Buuren S (2018). Flexible imputation of missing data.

[CR318] van der Horst MR, van der Lugt LM (2011). Coordination mechanisms in improving hinterland accessibility: Empirical analysis in the port of Rotterdam. Maritime Policy & Management.

[CR319] van der Horst MR, van der Lugt LM (2014). An Institutional Analysis of Coordination in Liberalized Port-related Railway Chains: An Application to the Port of Rotterdam. Transport Reviews.

[CR320] van Gelder, S. (2020). *The specifics of BRUcloud as a real live example of a Cargo Cloud*. http://www.clusters20.eu/wp-content/uploads/2020/07/20200630-3.-BRUcloud-Clusters_compressed-1.pdf. Accessed 22 Dec 2020.

[CR321] Vargo, S. L., & Lusch, R. F. (2008). Service-dominant logic: Continuing the evolution. *Journal of the Academy of Marketing Science,**36*(1), 1–10. 10.1007/s11747-007-0069-6

[CR322] Vieira, G. B. B., da Silva, R. M., Neto, F. J. K., dos Santos Senna, L. A., & Mulinas, A. M. (2014). Port governance model by managers’ and customers’ point of view: A study at port of Valencia, Spain. *International Business Research,**7*(8), 1. 10.5539/ibr.v7n8p1

[CR323] Vom Brocke, J., Simons, A., Niehaves, B., Niehaves, B., Reimer, K., Plattfaut, R., & Cleven, A. (2009). *Reconstructing the giant: On the importance of rigour in documenting the literature search process*. In S. Newell, E. Whitley, N. Pouloudi, J. Wareham, & L. Mathiassen (Eds.), (pp. 2206–2217). Presented at the *17th European Conference on Information Systems (ECIS) 2009*, Verona: Università di Verona, Facoltà di Economia, Departimento de Economia Aziendale. http://aisel.aisnet.org/ecis2009/372/. Accessed 12 Mar 2021.

[CR324] Wabtec Corp. (2020). *Port Optimizer*. https://www.wabtec.com/uploads/pdf/WAB_PortOptimizer_OnePager_v4.pdf. Accessed 30 Dec 2020.

[CR325] Wallbach S, Coleman K, Elbert R, Benlian A (2019). Multi-sided platform diffusion in competitive B2B networks: Inhibiting factors and their impact on network effects. Electronic Markets.

[CR326] Wang, Y., Miller, D. J., & Clarke, R. (2008). Approaches to working in high-dimensional data spaces: Gene expression microarrays. *British Journal of Cancer,**98*(6), 1023–1028. 10.1038/sj.bjc.6604207 PMC2275474 18283324

[CR327] Ward JH (1963). Hierarchical grouping to optimize an objective function. Journal of the American Statistical Association.

[CR328] Warren, T. (2021). *Microsoft is bringing Android apps to Windows 11 with Amazon’s Appstore.* The Verge. https://www.theverge.com/2021/6/24/22548428/microsoft-windows-11-android-apps-support-amazon-store. Accessed 21 Dec 2020.

[CR329] Warrick, J., & Nakashima, E. (2020). *Officials: Israel linked to a disruptive cyberattack on Iranian port facility*. Washington Post. https://www.washingtonpost.com/national-security/officials-israel-linked-to-a-disruptive-cyberattack-on-iranian-port-facility/2020/05/18/9d1da866–9942–11ea-89fd-28fb313d1886_story.html. Accessed 10 Dec 2020.

[CR330] Waterschoot, K. (2011). *Antwerp Port System*. WCO, Seattle. http://www.wcoomd.org/-/media/wco/public/global/pdf/events/2011/it/day-3/kristof_waterschoot.pdf?la=en. Accessed 14 Dec 2020.

[CR331] Weking J, Stöcker M, Kowalkiewicz M, Böhm M, Krcmar H (2020). Leveraging industry 4.0 – A business model pattern framework. International Journal of Production Economics.

[CR332] Wiech, S. (2019). *RheinPortsInformation System (RPIS)*. Meeting of the Commission Expert group on DINA, Brussels. https://ec.europa.eu/transparency/regexpert/?do=groupDetail.groupMeetingDoc&docid=29886. Accessed 11 Dec 2020.

[CR333] World Bank Group. (2015). *The Republic of Benin—Diagnostic Trade Integration Study (DTIS) Update:From rents to competitiveness* (Final Report No. 97242-BJ; Trade and Competitiveness Global PracticeAfrica Region, p. 156). World Bank. http://documents1.worldbank.org/curated/fr/759931468189257561/pdf/97242-ENGLISH-WP-P145228-PUBLIC-Box393236B-EV-final-Benin-DTISU-English-2015-10-30.pdf. Accessed 09 Dec 2020.

[CR334] WorldCargo News. (2018). Towards a US-wide data portal. *WorldCargo News*, 28. https://www.worldcargonews.com/in-depth/in-depth/towards-a-us-wide-data-portal. Accessed 30 Nov 2020.

[CR335] Woywod, K. (2015). *Mit Abfertigern und Spediteuren im Gespräch, DAKOSY Datenkommunikationssystem AG, Pressemitteilung—PresseBox*. Pressebox. https://www.pressebox.de/pressemitteilung/dakosy-datenkommunikationssystem-ag/Mit-Abfertigern-und-Spediteuren-im-Gespraech/boxid/729294. Accessed 22 Nov 2020.

[CR336] Wulf, L. D. (2004). *Tradenet in Ghana Best Practice of the Use of Information Technology* (Background Paper Prepared for the World Development Report 2005, p. 21).

[CR337] Wyciwski, M. (2016). *Launch of S)ONE Port Community System in Jamaica*. SOGET. https://ipcsa.international/armoury/resources/20161018prtransshipmentjamaicaen-1.pdf. Accessed 11 Dec 2020.

[CR338] Young Park, J., & Ik Yun, Y. (2018). *Global Logistics IT Leader—KL-Net*. UNESCAP. https://www.unescap.org/sites/default/files/4_KL-Net.pdf. Accessed 15 Mar 2021.

